# The selective PI3Kα inhibitor BYL719 as a novel therapeutic option for neuroendocrine tumors: Results from multiple cell line models

**DOI:** 10.1371/journal.pone.0182852

**Published:** 2017-08-11

**Authors:** Svenja Nölting, Jakob Rentsch, Helma Freitag, Katharina Detjen, Franziska Briest, Markus Möbs, Victoria Weissmann, Britta Siegmund, Christoph J. Auernhammer, Elke Tatjana Aristizabal Prada, Michael Lauseker, Ashley Grossman, Samantha Exner, Christian Fischer, Carsten Grötzinger, Jörg Schrader, Patricia Grabowski, on behalf of the GERMAN NET-Z study group

**Affiliations:** 1 Department of Internal Medicine II, Klinikum der Universität München (KUM), Ludwig-Maximilians-Universität München, München, Bavaria, Germany; 2 Department of Internal Medicine IV, Klinikum der Universität München (KUM), Ludwig-Maximilians-Universität München, München, Bavaria, Germany; 3 Dept. of Gastroenterology, CC13 (CBF and CVK), Charité - Universitätsmedizin Berlin, Berlin, Germany; 4 Dept. of Gastroenterology and Endocrinology, Zentralklinik Bad Berka GmbH, Bad Berka, Germany; 5 Dept. of Chemistry and Biochemistry, Freie Universität (FU) Berlin, Berlin, Germany; 6 Institute of Pathology, Charité - Universitätsmedizin Berlin, Berlin, Germany; 7 Department of General, Visceral and Thoracic Surgery, University Medical Center Hamburg-Eppendorf, Hamburg, Germany; 8 Institute for Medical Information Sciences, Biometry, and Epidemiology, Ludwig-Maximilians-Universität München, München, Bavaria, Germany; 9 Oxford Centre for Diabetes, Endocrinology and Metabolism, University of Oxford, Oxford, United Kingdom and Neuroendocrine Tumour Centre, Royal Free Hospital, London, United Kingdom; 10 Medical Department I, University Medical Center Hamburg-Eppendorf, Hamburg, Germany; University of South Alabama Mitchell Cancer Institute, UNITED STATES

## Abstract

**Background/Aims:**

The therapeutic options for metastatic neuroendocrine tumors (NETs) are limited. As PI3K signaling is often activated in NETs, we have assessed the effects of selective PI3Kp110α inhibition by the novel agent BYL719 on cell viability, colony formation, apoptosis, cell cycle, signaling pathways, differentiation and secretion in pancreatic (BON-1, QGP-1) and pulmonary (H727) NET cell lines.

**Methods:**

Cell viability was investigated by WST-1 assay, colony formation by clonogenic assay, apoptosis by caspase3/7 assay, the cell cycle by FACS, cell signaling by Western blot analysis, expression of chromogranin A and somatostatin receptors 1/2/5 by RT-qPCR, and chromogranin A secretion by ELISA.

**Results:**

BYL719 dose-dependently decreased cell viability and colony formation with the highest sensitivity in BON-1, followed by H727, and lowest sensitivity in QGP-1 cells. BYL719 induced apoptosis and G0/G1 cell cycle arrest associated with increased p27 expression. Western blots showed inhibition of PI3K downstream targets to a varying degree in the different cell lines, but IGF1R activation. The most sensitive BON-1 cells displayed a significant, and H727 cells a non-significant, GSK3 inhibition after BYL719 treatment, but these effects do not appear to be mediated through the IGF1R. In contrast, the most resistant QGP-1 cells showed no GSK3 inhibition, but a modest *activation*, which would partially counteract the other anti-proliferative effects. Accordingly, BYL719 enhanced neuroendocrine differentiation with the strongest effect in BON-1, followed by H727 cells indicated by induction of chromogranin A and somatostatin receptor 1/2 mRNA-synthesis, but not in QGP-1 cells. In BON-1 and QGP-1 cells, the BYL719/everolimus combination was synergistic through simultaneous AKT/mTORC1 inhibition, and significantly increased somatostatin receptor 2 transcription compared to each drug separately.

**Conclusion:**

Our results suggest that the agent BYL719 could be a novel therapeutic approach to the treatment of NETs that may sensitize NET cells to somatostatin analogs, and that if there is resistance to its action this may be overcome by combination with everolimus.

## Introduction

Neuroendocrine tumors (NET) are a heterogeneous group of malignancies. The therapy of choice for limited non-metastatic disease is surgery, but therapeutic options of metastatic disease are limited, as recently reviewed [1]. Low to intermediate grade metastatic NET may be managed by active surveillance, somatostatin analogs, loco-regional therapy, peptide receptor radionuclide therapy (PRRT), chemotherapy or molecular targeted therapy. If they progress and become more aggressive, chemotherapy shows only limited success (reviewed in [1]). However, several important molecular pathways involved in the pathogenesis and progression of NET have been identified to date, and open up new avenues for targeted therapies, including the tyrosine kinase inhibitor (TKI) sunitinib [2] or the mTORC1 inhibitor everolimus [3–5].

Besides the RAS/RAF/MEK/ERK or JNK pathways, one of the central signaling pathways is the phosphatidyl-inositol-3 kinase (PI3K)/AKT/mTOR sequence, which is frequently over-activated in NETs [6–14].

**Fig 1:**
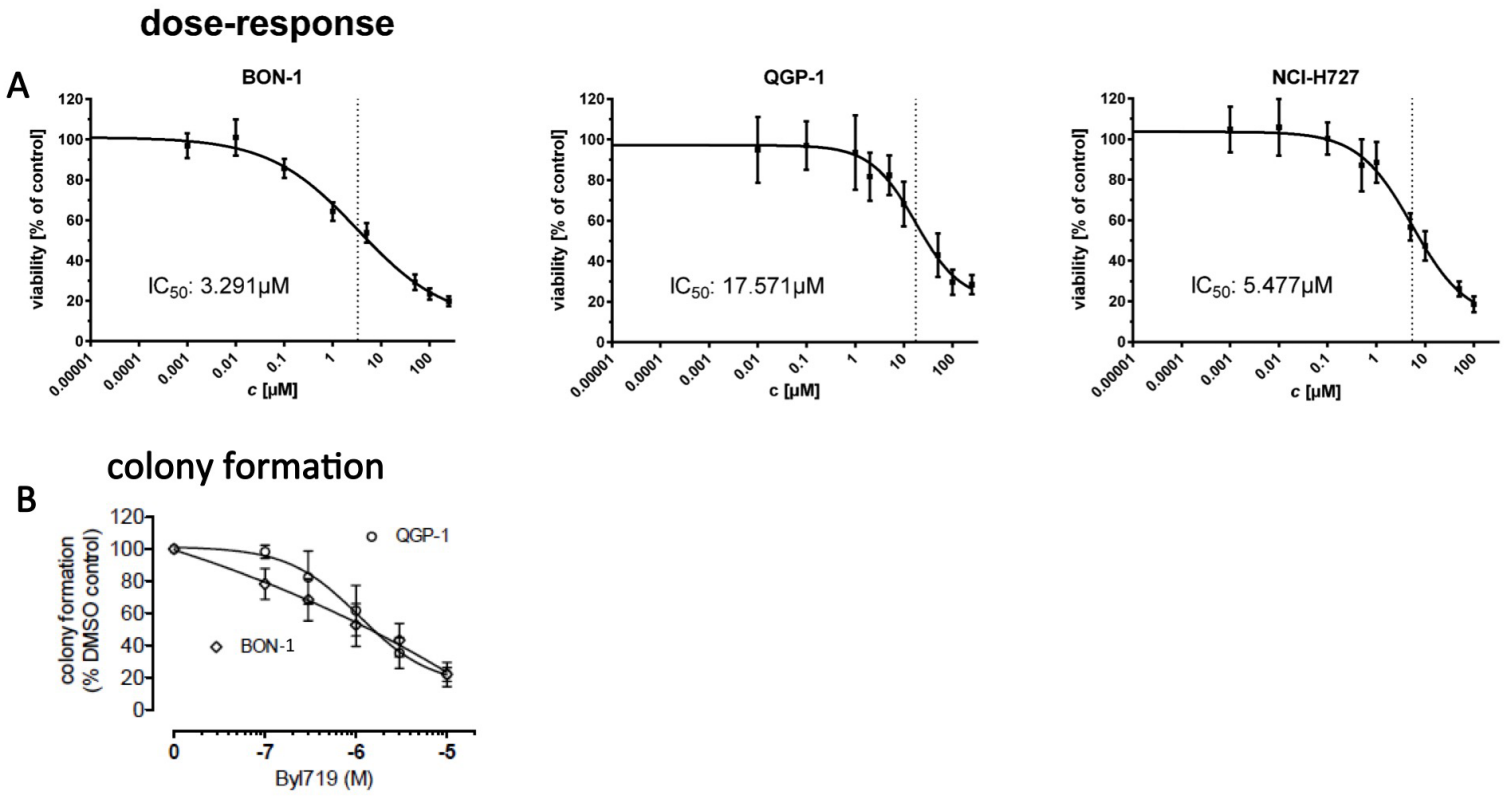
A: We treated cell lines with 9 different concentrations of BYL719 or vehicle control for 96 h and assessed cell viability with the WST-1 assay (Roche). Viability of treated cells was stated in percent vs. control (mean ± standard deviation (SD)). Dose-response curves and IC_50_ were calculated with GraphPad Prism software. B: In BON-1 and QGP-1 cells, colony formation was dose dependently reduced down to 22% of vehicel controls, with IC50 values of 1,3 μM and 1,8 μM, respectively. Data are presented as mean±SD of at least three independent experiments with five replicates per data point.

In brief, PI3K—encoded by the *PI3KCA* gene—is activated by different receptor tyrosine kinases (such as IGFR, EGFR, VEGFR, FGFR, RET) and in turn activates AKT which leads to inhibition of TSC1/2 and consequently to disinhibition/activation of *mTORC1/p70S6K*, phosphorylation of 4EBP1, activation of eIF-4E, cell proliferation and tumor growth (analogous to RAS/RAF/MEK/ERK signaling). RAS and PI3K may activate each other. Accordingly, the mTORC1 inhibitor everolimus has shown significant efficacy in NETs *in vitro*, *in vivo* and in clinical studies [1, 6], and has been approved for the treatment of pancreatic [5] and, very recently, of gastrointestinal and lung NETs [3, 4]. However, mTORC1 inhibition leads to a compensatory activation of PI3K/AKT signaling via p70S6K and IRS-1 activation, associated with an increase in pAKT^T308^ (via PI3K/PDK1), pAKT^S473^ (via mTORC2) and RAS/RAF/MEK/ERK signaling [15–19] (Fig 1). This compensatory up-regulation of PI3K/AKT and RAS/RAF/MEK/ERK may cause tumor cell resistance of initially sensitive cells [18–26]. Thus, it could be speculated that PI3K inhibitors working more upstream may bypass this mechanism of resistance and be more effective than mTORC1-inhibition alone. Indeed, different panPI3K inhibitors (LY294002, BKM120) or the dual PI3K/mTORC1/2 inhibitor BEZ235 alone and in combination with mTORC1 inhibitors and a MEK inhibitor, respectively, have shown anti-tumor potential in NET cells *in vitro* and *in vivo* [17, 18, 27–31]. In pancreatic NET cells, a synergistic effect of everolimus plus BEZ235 has been reported [18]. However, global clinical development of BEZ235—the most effective agent in pancreatic NET cells *in vitro* [18]—has been terminated, and two clinical trials in pancreatic NETs were stopped early due to poor tolerability, frequent treatment discontinuation, a non-fulfilled statistical endpoint and, moreover, no clear superiority to everolimus [24]. However, selective inhibition of the PI3Kα/110α-subunit—the predominant catalytic isoform in neuroendocrine pancreatic β-cells [32] involved in glucose homeostasis regulation [33, 34] and angiogenesis [35]—may be more effective and have a better safety profile than panPI3K inhibitors.

BYL719 is a selective PI3Kα inhibitor [36, 37] which has shown anti-proliferative activity in the pancreatic NET cell lines BON-1 and QGP-1 in a recent study [18]. BON-1 cells were more sensitive to BYL719 than QGP-1 cells in that study [18]. Notably, in pancreatic NET cells, the selective PI3Kα inhibitor BYL719 led to an additional weak inhibition of mTORC1 at 10 μM; in contrast, the panPI3K inhibitor BKM120 did not induce supplementary mTORC1 inhibition. BKM120 plus everolimus was not synergistic, whereas BYL719 plus everolimus was not tested in NET cells [18].

On the basis of that previous study, which showed anti-proliferative effects and partial PI3K/mTORC1 inhibition in BON-1 and QGP-1 cells after BYL719 treatment [18], we have now investigated the effects of the selective PI3Kα inhibitor BYL719 on pancreatic (BON-1, QGP-1) and lung (H727) NET cells in detail. Besides exploring its influence on cell viability, colony formation and PI3K/AKT/mTORC1/2-signaling, we also investigated the effects on other signaling pathways, such as IGF1 and GSK3, assessing compensatory signaling pathway activations and downstream targets; these included apoptosis, cell cycle, cell differentiation [somatostatin receptor (SSTR)1/2/5 expression], chromogranin A (CgA) expression and secretion. Moreover, we tested the combination treatment of BYL719 with everolimus in NET cell lines, since additional mTORC1 inhibition has been shown to enhance sensitivity to BYL719 in breast cancer cells [38]. The aim of our study was to assess the efficacy of the PI(3)Kα inhibitor BYL719 in different NET cell lines and to determine the underlying mechanisms of action in order to explain the different cell line sensitivities, and to see if we could overcome partial resistance via combination treatments. We hypothesized that BYL719 might be effective at inhibiting the proliferation of NET cells to a different extent depending on cell line, and that partial resistance may be overcome by combination treatments.

Our results showed for the first time that the selective PI3Kα or BYL719 led to GSK3 inhibition and induction of re-differentiation with a significant increase of SSTR1/2 transcription in pancreatic BON-1 and pulmonary H727 cells associated with higher efficacy of BYL719 and higher cell line sensitivity, compared to QGP-1 cells. In contrast, the lower sensitivity of QGP-1 cells to BYL719 was associated with absent GSK3 inhibition and an absence of re-differentiation; partial resistance to BYL719 could be overcome by combination with everolimus via simultaneous PI3K/mTORC1 inhibition and hence strongly increased SSTR2 transcription. Thus, BYL719 might be an effective therapy for NETs, especially in combination with everolimus, and might sensitize NET cells to somatostatin analogs.

This study was a collaborative effort of five German research groups with complementary expertise in preclinical studies on NET cell lines. The long-term goal is the establishment of a preclinical collaborative network (NET-Z) that would provide an efficient and fast preclinical evaluation of novel drug candidates for clinical trials.

## Materials and methods

### Cell lines and drugs

Pancreatic neuroendocrine tumor cell lines BON-1 [39] and QGP-1 [40] (both obtained from Japanese Collection of Research Bioresources) and the typical bronchial carcinoid-NET cell line NCI-H727 (H727) [41] (purchased from ATCC, Manassas, VA) underwent authentication at the DSMZ (Braunschweig, Germany, in 2014), and their neuroendocrine features were confirmed by immunocytochemistry. Participating researchers received cell aliquots of the same passage together with SOPs for culture procedures. Cell lines had been tested for *PIK3CA* mutations by panel sequencing (Ion Seq Torrent Lung and Colon Panel v2). No *PIK3CA* mutations had been detected. BYL719 is a selective PI3Kα inhibitor developed by Novartis Pharma. All cell lines have been treated with different concentrations (10 nM– 250 μM) of BYL719 (kindly provided by Novartis) versus DMSO control for several time periods according to assay type. Everolimus was purchased from Selleckchem (Munich, Germany) and used in concentrations between 1 and 10nM.

### Cell viability

For determination of cell viability, the WST-1 Assay (Roche) was utilized according to the manufacturer`s instructions followed by calculation of IC_50_ and IC_85_. The relative IC_50_ and IC_85_ values (indicating the concentration of half-maximal and 85% of maximal effect) were determined by non-linear regression using GraphPad Prism 6 software (mean of three independent experiments, 95% CI). Cells were incubated with 9 different concentrations of BYL719 (1nM-250μM) against vehicle control for 96h. The procedure of experiments and analysis of results were conducted as described previously [42]. For statistical analysis, we used Microsoft Excel and GraphPad Prism 6 analysis software (San Diego, Calif., USA). The data passed a Kolmogorov-Smirnov test (with Dallal-Wilkinson-Lilliefors p value) for a Gaussian distribution, appropriate for data sets below 20 data points. Outliers were identified by Grubbs' test with α = 0.05. Dose-response curves were fitted to the measured data using the method of least squares with variable slope; goodness of fit was quantified with R^2^ and sums of squares. For detection of significant differences between two or more data groups, we used ANOVA followed by the Dunnett`s test with a p value of <0.05. Data are presented as mean±standard deviation (SD) of at least three independent experiments with five replicates per data point.

For the combination experiments with BYL719/everolimus co-treatment, cell viability was assessed by “Cell Titer 96 Aqueous One Solution” MTS cell viability assay (Promega, Madison, WI, USA) according to the manufacturer`s instructions as previously described in detail [43]. Cells were treated with various concentrations of 1 μM—20 μM BYL719, 1 nM -10 nM everolimus, or a combination of both drugs for the indicated incubation times. The low dose combination experiments of 1 μM BYL719 plus 1 nM everolimus were conducted in Hamburg with an incubation period of 120 h. The higher dose experiments were conducted in Munich with an incubation period of 144 h. To identify potential synergistic or antagonistic effects between BYL719 and everolimus on the natural logarithm of cell viability (ln cell viability), we used Linear Mixed Effects Models. BYL179, everolimus and the interactions between both were considered as fixed effects, the trial as a random effect. If a significant positive interaction between BYL179 and everolimus on cell survival was found, we concluded an antagonistic effect; if a significant negative interaction on cell survival was found, we concluded a synergistic effect. One model was estimated for each cell line. Statistical significance was defined at p<0.05. All computations were performed with R 3.2.2. For each MTS experiment, 4–6 independent samples per data point have been analyzed; each experiment was repeated at least two times in an independent manner.

### Colony formation

Soft agar colony formation assays were used for assessment of anchorage-independent growth, as previously described [44]. Each experiment was repeated at least three times in an independent manner.

### Cell cycle

For flow cytometry analysis of the cell cycle, cells were seeded and incubated with two different concentrations of BYL719 (IC_50_ and IC_85_ of the cell viability experiments for each cell line) against vehicle control for 72h. Detailed conditions and procedure of treatment, staining, measurement and analysis of results are described before [42]. DNA was stained with propidium iodide and mitotic cells were detected with an antibody against phospho-Histon H3. For statistical analysis, we used Microsoft Excel and GraphPad Prism 6 analysis software (San Diego, Calif., USA). The data passed a Kolmogorov-Smirnov test (with Dallal-Wilkinson-Lilliefors p value) for a Gaussian distribution, appropriate for data sets below 20 data points. Outliers were identified by Grubbs’ test with α = 0.05. For detection of significant differences between two or more data groups, we used ANOVA followed by the Dunnett`s test with a p value of <0.05. Data are presented as percentage of all detected cells (about 50.000 cells) (mean ± SD) of at least four independent experiments.

### Apoptosis

Caspase activity/apoptotic activity was determined using the Apo-One homogeneous caspase-3/7- Assay kit (Promega, #G7790): 10.000 cells per well were seeded, grown for 24 h and incubated for 24 h with different concentrations of BYL719. Concentrations of 10 μM (effective dose close to the IC50 of the cell viability experiments) and higher doses were tested. Caspase-3/7 activity was assessed following the manufacturer’s instructions. For statistical analysis, *a priori* tests evaluating the normal distribution and homogeneity of variances were performed by the Kolmogorov-Smirnov-Test and the Levene′s Test of the SPSS statistical package SPSS (version 13.0 for Windows, SPSS Inc (2005), Chicago, USA). Non-parametric criteria were met; therefore, the Kruskal-Wallis followed by the Mann-Whitney test was performed. Statistical significance was assessed at p<0.05. Three independent samples per data point have been analyzed; each experiment was repeated at least two times in an independent manner. The results are displayed as mean ± SD.

### Western blot technology

Western blot experiments were performed as previously described [45]. In brief, BON-1, H727 and QGP-1 cells were grown in 6-well plates for 24 h in complete medium. Afterwards, the medium was replaced by fresh 10% FBS medium and the respective doses of BYL719, everolimus or NVP-AEW541 were added for the respective incubation periods. In case of IGF1 stimulation, cells were pre-treated with the respective drugs for 48 h followed by IGF1 stimulation (50 ng/ml) for 30 minutes. After being washed twice in PBS, cells were lysed in 200 μl lysis buffer (M-PER ^®^ Mammalian Protein Extraction Reagent containing HALT ^™^ protease & phosphatase inhibitor cocktail, Thermo Scientific, Rockford, USA). Lysates were centrifuged at 13,000 rpm for 10 min. Supernatants were adjusted to the same protein concentration (30–50 μg/50 μl) (Rotiquant Universal, Carl Roth, Karlsruhe, Germany). Equal amounts of protein were boiled for 5 minutes and denatured in Sodium dodecyl sulfate (SDS) sample buffer (0.25% Tris HCL, 40% glycerol, 2% SDS, 1% dithiothreitol, bromophenol blue, pH 8.8) before protein extracts were separated by SDS-PAGE and analysed by Western blot technology, as described before in detail [45].

The following primary antibodies were utilized: Primary monoclonal antibodies against pAKT (Ser473) (#4060), pAKT (Thr308) (#13038), AKT (#2920), pGSK3 (Ser21/9) (#8566), GSK3 (#5676), pERK1/2 (Thr202/Tyr204) (#4370), pp70S6K (Thr389) (#9234), p70S6K (#9202), pEGFR (Tyr1068) (#3777), EGFR (#4267), p4EBP1 (Ser65) (#9451), 4EBP1 (#9644), pS6 (Ser240/4) (#5364), S6 (#2317), p27 Kip1 (#3686), Caspase 3 (#9662), PARP (#9542), pChk1 (Ser345) (#2341), Chk1 (#2360), pRb (Ser780) (#9307), Cyclin D3 (#2936), pIGF1 (Tyr1131) (Tyr1146) (#3021), IGF1 (#3027) (all from Cell Signaling, Danvers, MA), Rb (#614602) (Biolegend, San Diego, USA), p21 Waf1/Cip1 (610233) (from BD Transduction Laboratories, Heidelberg, Germany), Actin (A5441) (Sigma, St. Louis, USA), Erk 1/2 (06–182) (Merck-Millipore, Darmstadt, Germany), SSTR2 (Abcam, Cambrdige, UK), GAPDH (Clone 6C5, Merck-Millipore, Darmstadt, Germany).

Actin was used as loading control. Peroxidase-conjugated polyclonal secondary antibody (1:25000) was incubated for 2 h, the blots were immersed in the chemiluminescent substrate SuperSignal West Dura (Thermo Scientific, Rockford, USA) and images were taken with an ECL Chemocam Imager (INTAS, Göttingen, Germany) or generated by using conventional X-ray films. Optical density of the approximately sized bands was densitometrically quantified using ImageJ 1.440 software (Wayne Rasband, National Institute of Health, USA). Band intensities were quantified from at least 3 independent experiments for each cell line and protein, and are described as the mean percentage relative to the untreated control (100%). We performed statistical analysis according to the following method: A linear mixed effects model was estimated for the logarithm of the relative increase [compared to the respective control group] with the trial as random effect. The logarithm was chosen to account for the multiplicative nature of the data. Analogously, means and standard deviations are reported as geometric means and geometric standard deviations of the relative increase, respectively. A geometric mean of "1.0" has to be interpreted as "equal to the control group" and for the geometric standard deviation “1.0” refers to “no variation“. We compared the phospho(p)-protein expression to the control to adequately assess the extent of activation or inhibition of signaling pathways after drug treatment and compare it between the different cell lines. All original uncropped Western blots are shown as supplementary Figures (S1–S23, S29 and S30 Figs).

### Gene expression analysis of somatostatin receptors (*SSTR) 1/2/5* and chromogranin A (*CHGA)*

For monotherapy with BYL719 we evaluated an effective dose close to the IC50 of the cell viability experiments (10μM). For combination treatment we used concentrations that gave us a synergistic effect at the lowest dose, therefore achieving maximum synergistic effects but minimizing potential toxic side effects. Thus, tumor cells were treated for 96h with BYL719 at 10μM (monotherapy) or at 1μM BYL719 and 1nM everolimus (combination treatment). Total RNA was isolated from cell cultures using the Nucleo Spin RNA, DNA and protein purification Kit (Macherey-Nagel, Düren, Germany) according to the manufacturer’s protocol. Total RNA was reverse-transcribed using the High Capacity cDNA Reverse Transcription Kit (Applied Biosystems, Foster City, CA) and a T3 Thermocycler (Biometra, Göttingen, Germany).

We used pre-validated TaqMan Primers for SSTR1, SSTR2, SSTR3, SSTR5 and GAPDH for real-time PCR. These primers were purchased from Applied Biosystems. Samples in duplicate were subjected to PCR in a Step One Plus Real-Time PCR System (Applied Biosystems). Values were expressed as (Ct) values normalized to housekeeping gene GAPDH using the 2^-ΔΔCt^ method. All graphs and statistical analysis were performed using GraphPad Prism 4 utilizing standard two-tailed unpaired Students t-test for normally distributed data. P-values <0.05 have been considered significant (*<0.05; **<0.005, ***<0.001). Data are presented as mean ± standard error of mean (SEM) of at least three independent experiments with three replicates per data point.

### ChromograninA (CgA) ELISA

BON, H727 and QGP-1 cells were seeded in 24-well plates at a density of 2x10^5^ per well and grown for 24 h. Cells were treated with either 0.1% DMSO (vehicle control), PDBu (Phorbol-12,13-dibutyrate; Sigma-Aldrich, Deisenhofen, Germany) as a positive control for the induction of secretion or BYL719 for 24 h in growth medium. Media were collected and remaining cell debris was eliminated by centrifugation for 3 min at 200 x *g*. CgA concentration in the supernatants was measured using CgAnalyze Kit (DAKO, Hamburg, Germany) according to the manufacturer's protocol. Two independent experiments with three replicates per data point were analyzed.

## Results

### Cell viability, apoptosis and cell cycle

#### Significant reduction cell viability in NET cell lines in vitro

The pancreatic NET cell lines BON-1 and QGP-1 and the typical bronchial carcinoid cell line H727 were treated with increasing doses of BYL719 for 96 h and cell viability was determined by the WST-1 assay. BON-1 cells showed the highest sensitivity to BYL719 with an IC_50_ of 3.29 μM, followed by H727 cells with an IC_50_ of 5.48 μM, and QGP-1 cells with the lowest sensitivity to BYL719 and an IC_50_ of 17.57 μM (Fig 1A).

The IC_85_ was 100 μM in all cell lines (Fig 1A): 96 h treatment with low doses of 1 μM BYL719 only led to a slight decrease in cell viability in all three cell lines (by 36%, 11% and 7% in BON-1, H727 and QGP-1 cells, respectively) (Fig 1A).

Colony formation assay demonstrated the efficacy of BYL719 in pancreatic NET cells with a higher sensitivity for BON-1 cells compared to QGP-1 cells. BYL719 dose-dependently reduced colony formation down to 22% of vehicle controls, with IC_50_ values of 1.3 μM (BON-1) and 1.8 μM (QGP-1) (Fig 1B). H727 cells did not form colonies.

#### Induction of apoptosis

**Fig 2:**
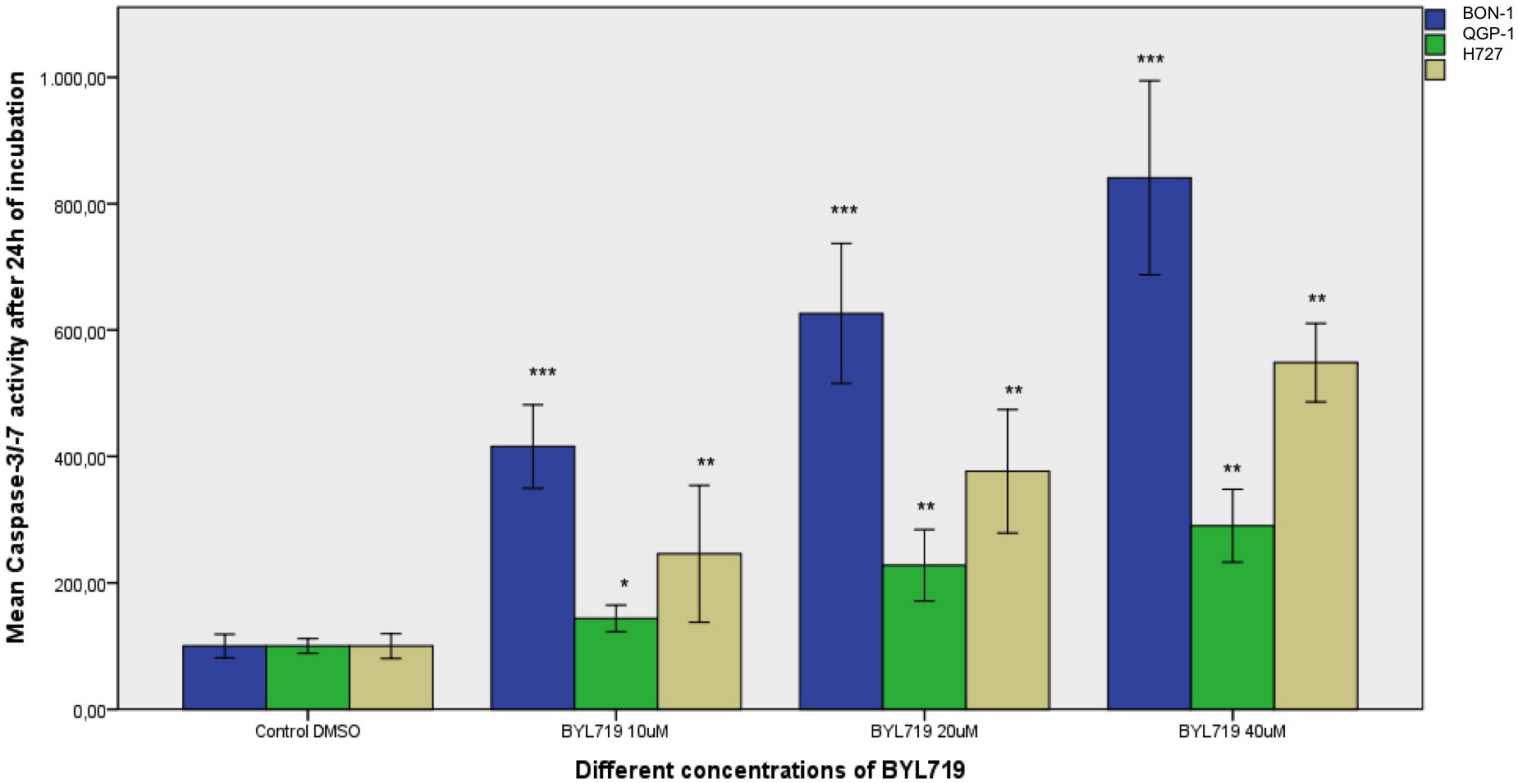
Assessment of apoptosis: Caspase 3/7 assay results after 24 h treatment with different doses of BYL719 are shown as mean percentage of caspase 3/7 activity referred to the untreated control (100%) ± SD: Strongest apoptosis was induced in BON-1 cells, followed by H727 cells. QGP-1 cells showed the least but still significant apoptosis. * p<0.05; ** p≤0.01; *** p≤0.001 (two independent experiments with three replicates per data point).

Caspase 3 and 7 activity was measured by caspase3/7 assay after 24 h of treatment with increasing concentrations of BYL719: 10 μM and higher concentrations of BYL719 significantly induced apoptosis in all 3 cell lines, BON-1, QGP-1 and H727 cells. The strongest apoptosis induction was found in BON-1 cells, followed by H727 cells, with QGP-1 showing the least but still significant apoptosis. The data shown represent the mean ± SD (Fig 2).

**Fig 3:**
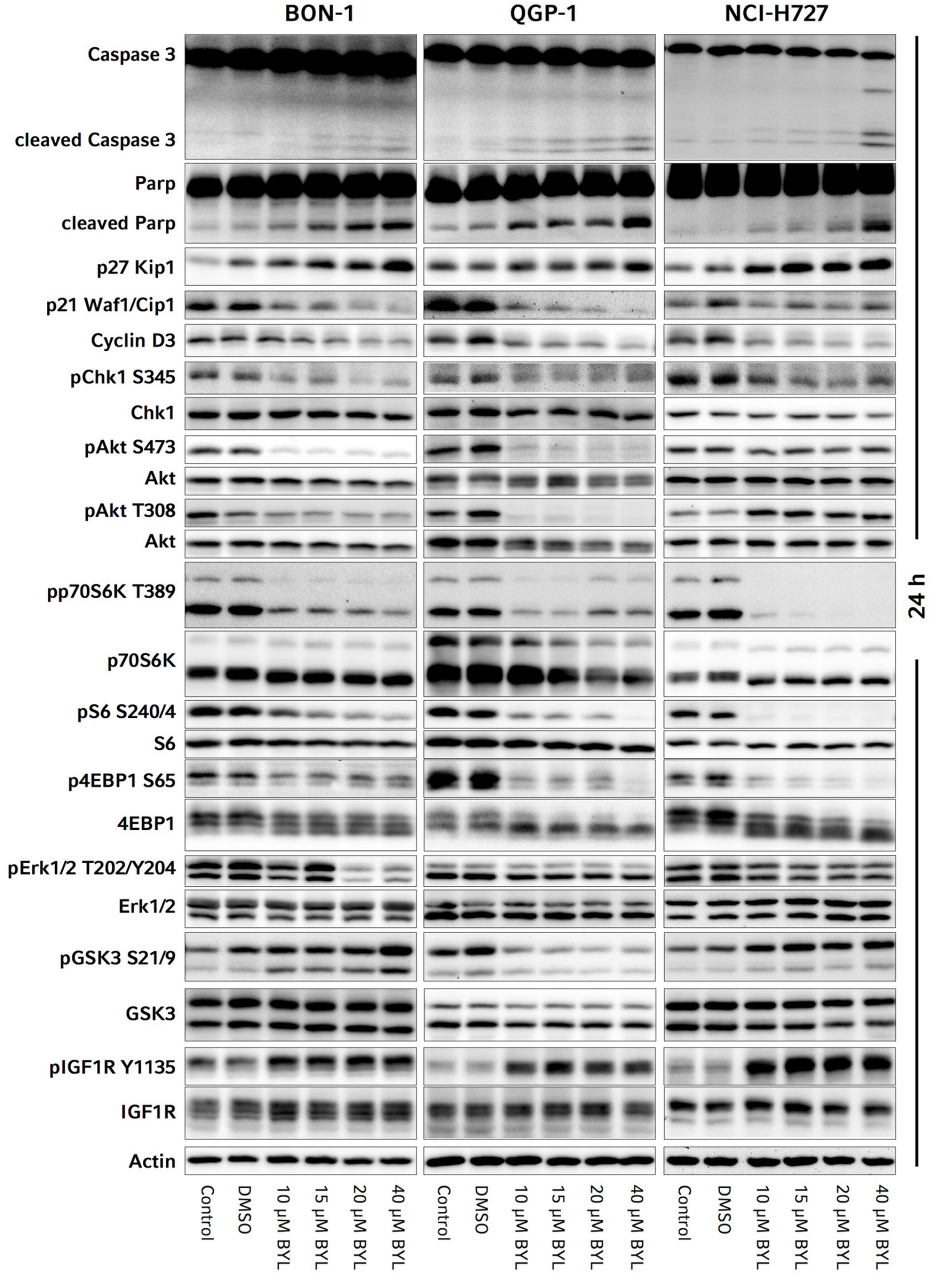
Representative western blots of the investigated signaling pathways, apoptosis and cell cycle markers in BON-1, QGP-1 and H727 cells: The means, standard deviations and p-values of the western blot quantification and statistical analysis from at least three independent replicates are given in S1 Table.

Consistent with clear apoptosis induction, western blot analyses showed a dose-dependent significant increase of cleaved caspase-3 and cleaved PARP in all three cell lines after 24 h of treatment with BYL719 (S1 Table, Fig 3).

#### Cell cycle arrest in G_0_/G_1_

While p27 and p21 inhibit cell cycle progression by inhibition of cyclin dependent kinases, Cyclin D3 induces cell cycle progression from G_0_/G_1-_ to S-phase,. Activation of the DNA-damage checkpoint marker CHK1 leads to cell cycle arrest, DNA-repair and if ineffective cell death to prevent damaged cells from cell cycle progression.

**Fig 4:**
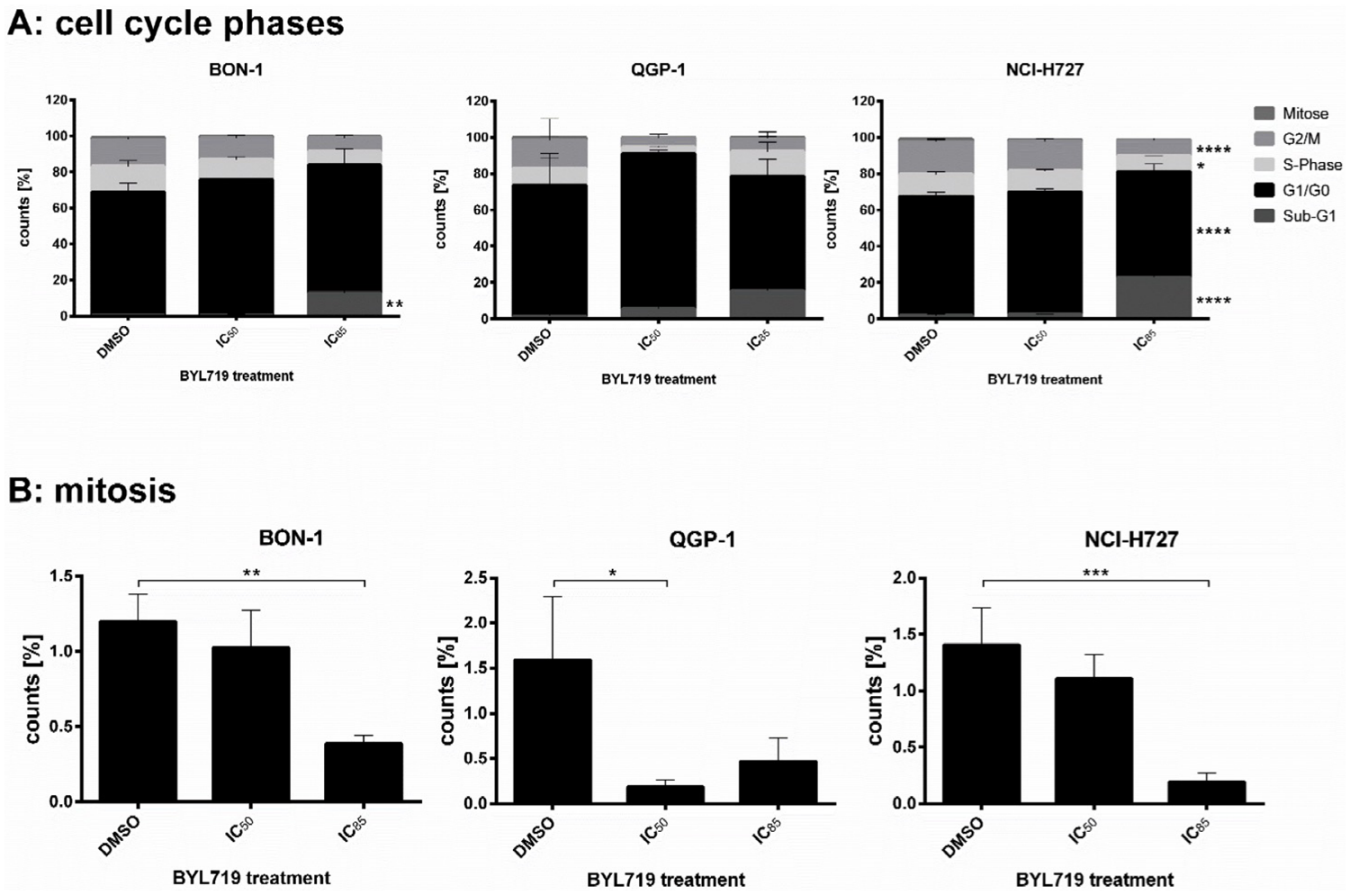
Cell lines were treated with BYL719 (IC_50_ and IC_85_) for 72 h. Cells were stained with PI (DNA content) analysis solely included and mitosis-specific Phospho(Ser10)-Histone H3 immunostain followed by flow cytometry assessment (single cells). According to the stain, events were divided into cells of several cell cycle phases or were classified as sub-G1-events indicating cell death (FlowJo software), given in percent of all detected cells (mean ± SD) of at least four independent experiments. A: Cell cycle. B: Mitotic index. * p<0.05; ** p≤0.01; *** p≤0.001; **** p≤0.0001.

BON-1, QGP-1 and H727 cells were treated with BYL719 for 72 h and changes in cell cycle patterns were assessed by flow cytometry. BYL719 increased the G_0_/G_1_ fraction at IC_50_ concentrations indicating induction of G_0_/G_1_ cell cycle arrest. At higher doses (IC_85_), a sub-G1 fraction suggested induction of cell death (Fig 4A).

Furthermore, the percentage of mitotic cells was assessed by mitotic index flow cytometry (mitotic index: percentage of cells which are in the mitotic phase). In all three cell lines the percentage of mitotic cells decreased at high dose of BYL719 treatment (mean of four independent experiments, including standard deviation) (Fig 4B).

Consistent with induction of G_0_/G_1_-cell cycle arrest, p27 was significantly increased after 24 h treatment in all three cell lines (S1 Table, Fig 3); the strongest up-regulation of p27 at 10–20 μM BYL719 was found in BON-1 cells (S1 Table, Fig 3). Moreover, there was a non-significant decrease of Cyclin D3 in all three cell lines, also promoting G_0_/G_1_-cell cycle arrest (S1 Table, Fig 3). According to the induction of apoptosis, as discussed below, there was a significant reduction of the cell cycle regulator p21 in all three cell lines after 24 h treatment (S1 Table, Fig 3).

We observed a significant inhibition of the DNA damage checkpoint marker Chk1 (pChk1^Ser345^) after 24 h treatment in all three cell lines—in BON-1 cells at doses higher than 15 μM, in H727 and QGP-1 cells at all tested doses (S1 Table, Fig 3).

### Signaling pathways

#### PI3K/AKT/mTORC1/2 and ERK signaling: Partial inhibition of PI3K/AKT^Ser473/Thr308^, mTORC1 and ERK signaling

Activated/phosphorylated (p)AKT^Ser473^ indicates mTORC2-dependent PI3K/AKT activity, while pAKT^Thr308^, which is mediated by PDK1, is a marker PI3K/PDK1/AKT activity; pp70S6K, pS6 and p4EBP1 are downstream-targets of mTORC1 and markers of mTORC1 activation; pERK displays activation of RAS/RAF/ERK signaling.

In BON-1 and QGP-1 cells, there was a significant inhibition of AKT^Ser473^ at all tested doses (10–40 μM) of BYL719 after 24 h treatment; a lower and inconsistent inhibition of PI3K/AKT^Ser473^ was seen in H727 cells with a statistical significance at doses higher than 15 μM (S1 Table, Fig 3). Similar results were found for PI3K/AKT^Thr308^ with a significant inhibition in QGP-1 cells at all tested doses and in BON-1 cells at doses higher than 15 μM. In contrast, in H727 cells there was a non-significant activation of PI3K/AKT^Thr308^ (S1 Table, Fig 3). In all three cell lines, mTORC1 was significantly inhibited after 24 h treatment with 10–40 μM BYL719 as demonstrated by significant reduction of pp70S6K, pS6 and p4EBP1 (S1 Table, Fig 3). The strongest decrease of pp70S6K and pS6 was found in H727 cells, followed by QGP-1 cells, and the lowest, but a significant decrease was still observed in BON-1 cells. The decrease of p4EBP1 was significant in H727 cells at all tested doses, but in BON-1 and QGP-1 cells only at the highest tested dose (S1 Table, Fig 3); We observed a slight inhibition of ERK signaling upon 24 h BYL719 treatment in all three cell lines with a statistical significance only at high doses (at 20 μM in BON-1 and 40 μM in QGP-1 and H727 cells) (S1 Table, Fig 3).

#### Inhibition of GSK3 in BON-1 and H727 cells, but not in QGP-1 cells

The growth-factor-induced PI3K/AKT signaling pathway phosphorylates and thereby inhibits GSK3 [46–48]. In our study GSK3α and β (GSK3) behaved similarly. We quantified both together and separately (S1 Table). Unexpectedly, despite significant PI3K/AKT inhibition after 24 h BYL719 treatment, there was a dose-dependent phosphorylation/inhibition of GSK3 in BON-1 and H727 cells with a significant inhibition of GSKα/β and especially GSK3 in BON-1 cells at doses higher than 10 μM (S1 Table, Fig 3); in QGP-1 cells, there was no inhibition, but a modest activation of GSK3 at doses higher than 10 μM BYL719 after 24 h treatment (S1 Table, Fig 3). Additional Western blot analysis with low dose treatment of 1 μM BYL719 was performed in the most sensitive cell line BON-1 as our cell line model: we showed no effects on pGSK3, compared to the control (S24 Fig). PGSK3 was increased in BON-1 cells at 2.5 μM BYL719 and higher doses (S25 Fig).

#### Receptor tyrosine kinases: Strong activation of IGF1R signaling

Phosphorylated (p)IGF1R and pEGFR are markers of activated receptor tyrosine kinases IGF1R and EGFR, respectively. IGF1R may also phosphorylate/inhibit GSK3 [46–48]. In BON-1, QGP-1 and H727 cells, IGF1R was significantly activated after treatment with 10–40 μM BYL719 for 24 h with strongest activation in H727 cells, followed by BON-1 cells and lowest activation in QGP-1 cells (S1 Table, Fig 3). Additional Western blot analysis after low dose treatment with 1 μM BYL719 still showed IGF1R activation in BON-1 cells as our cell line model (S24 Fig).

EGFR activity was not notably altered in any cell line.

#### Combination treatment of BYL719 with the specific IGF1R inhibitor NVP-AEW541

In order to prove a potential dependence of GSK3 phosphorylation on IGF1R activation in response to BYL719 treatment, we tested co-treatment of BYL719 with the specific IGF1R inhibitor NVP-AEW541 in all three cell lines for an incubation period of 48 h; surprisingly, such co-treatment showed at least additive inhibitory effects on BON-1, H727 and QGP-1 cell viability. We confirmed again that BYL719 led to phosphorylation/inhibition of GSK3 in BON-1 and H727 cells. However, unexpectedly, this inhibitory effect on GSK3 was even enhanced in both BON-1 and H727 cells by co-treatment with NVP-AEW541 (and not blocked), being accompanied by stronger IGF1R phosphorylation/activation after combination treatment. In QGP-1 cells neither drug alone nor the combination changed GSK3 phosphorylation: in QGP-1 cells NVP-AEW541 alone inhibited IGF1R phosphorylation/activation and co-treatment inhibited the BYL719-induced IGF1R activation. In QGP-1 and H727 cells total IGF1R expression was slightly increased after NVP-AEW541 treatment alone. H727 cells showed a low baseline phosphorylation of the IGF1R. In BON-1 cells, which showed a very low baseline phosphorylation/activation of IGF1R, NVP-AEW541 did not change phosphorylation or total expression levels of IGF1R (S26 Fig).

The low baseline phosphorylation/activity of the IGF1R in BON1 and H727 cells might not have been sufficient to show the effects of specific IGF1R inhibition by NVP-AEW541; moreover, others have shown a dependence of GSK3 phosphorylation on IGF1R activity in medulloblastoma cells [49], but they stimulated their cells with IGF1 to demonstrate clear results. We therefore enhanced and optimized our experimental design by using IGF1 stimulation according to the previously-published experimental design [49]. We used a slightly lower dose of 10 μM BYL719 because we expected a much stronger IGF1R phosphorylation after IGF1 stimulation, and we wished to investigate if the absent effect of NVP-AEW541 on BYL719-induced GSK3 phosphorylation could also be observed at lower doses of 10 μM BYL719. We could then show strong baseline IGF1R phosphorylation/activity after IGF1 stimulation in all three cell lines, which could be clearly inhibited by NVP-AEW541 in all three cell lines. As expected, BYL719 led to strong IGF1R activation in all cell lines, and NVP-AEW541 prevented the BYL719-induced IGF1R activation. However, while the baseline phosphorylation of GSK3 increased after stimulation with IGF1 in all three cell lines, it was even more strongly increased after either NVP-AEW541 treatment or BYL719 treatment alone in BON-1 (especially GSK3β) and H727 cells. In QGP-1 cells no changes in GSK3 phosphorylation after drug treatment were seen, consistent with our previous results. Thus, despite IGF1R inhibition after combination treatment in all three cell lines, the phosphorylation/inhibition of GSK3 was even stronger after the combination treatment in H727 cells and not changed in BON-1 and QGP-1 cells compared to single BYL719 treatment (S27 Fig). Therefore, blockade of the IGF1R did not attenuate BYL719-induced GSK3 phosphorylation refuting our initial hypothesis of the dependence of BYL719-induced GSK3 phosphorylation on IGF1R activation in BON1 and H727 cells.

### mRNA expression of NET markers *(SSTR1*, *SSTR2*, *SSTR5*, *CHGA)*: Induction of cell re-differentiation in BON-1 and H727 cells, but not in QGP-1 cells

**Fig 5:**
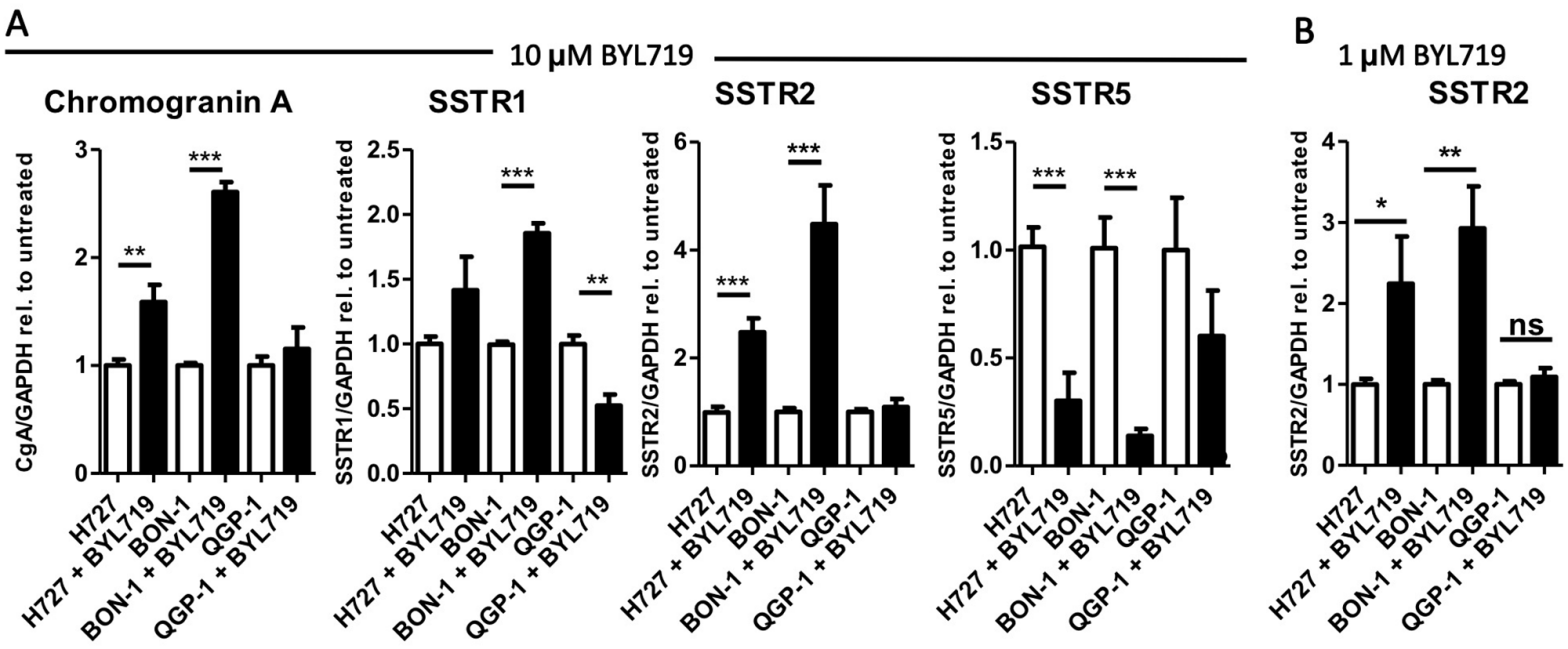
Neuroendocrine marker expression was increased by BYL719 treatment. A: BON-1, H727 and QGP-1 cells were treated with 10 μM BYL719 for 96 h and mRNA expression of Chromogranin A *(CHGA)*, *SSTR1*, *SSTR2* and *SSTR5* was analyzed by qPCR. B: BON-1, H727 and QGP-1 cells were treated with 1 μM BYL719 for 96 h and mRNA expression of *SSTR2* was analyzed by qPCR; Values are summarized from three independent experiments of three replicates per data point. * p<0.05; ** p≤0.01; *** p≤0.001.

Treatment of cells with 10 μM BYL719 for 96 h induced mRNA expression of genes related to neuroendocrine differentiation in BON-1 and H727 cells. Transcripts for CgA (*CHGA*), *SSTR1* and *SSTR2* were induced in H727 and BON-1 cells, whereas *SSTR5* was reduced. In particular, we observed a 2.5- (CI 1.8–3.1) and 4.5- (CI 2.6–6.3) fold induction of *SSTR2* mRNA upon BYL719 treatment in H727 and BON-1 cell, respectively (p<0.001). In contrast, in QGP-1 cells *SSTR1* transcripts were reduced, while *CHGA*, *SSTR2* and *SSTR5* were unaffected (Fig 5A). We also tested SSTR2 mRNA expression in all three cell lines after treatment with a low dose of 1 μM BYL719 for 96 h and found significant SSTR2 induction in BON-1 and H727 cells, but not in QGP-1 cells.

### CgA secretion was not altered

**Fig 6:**
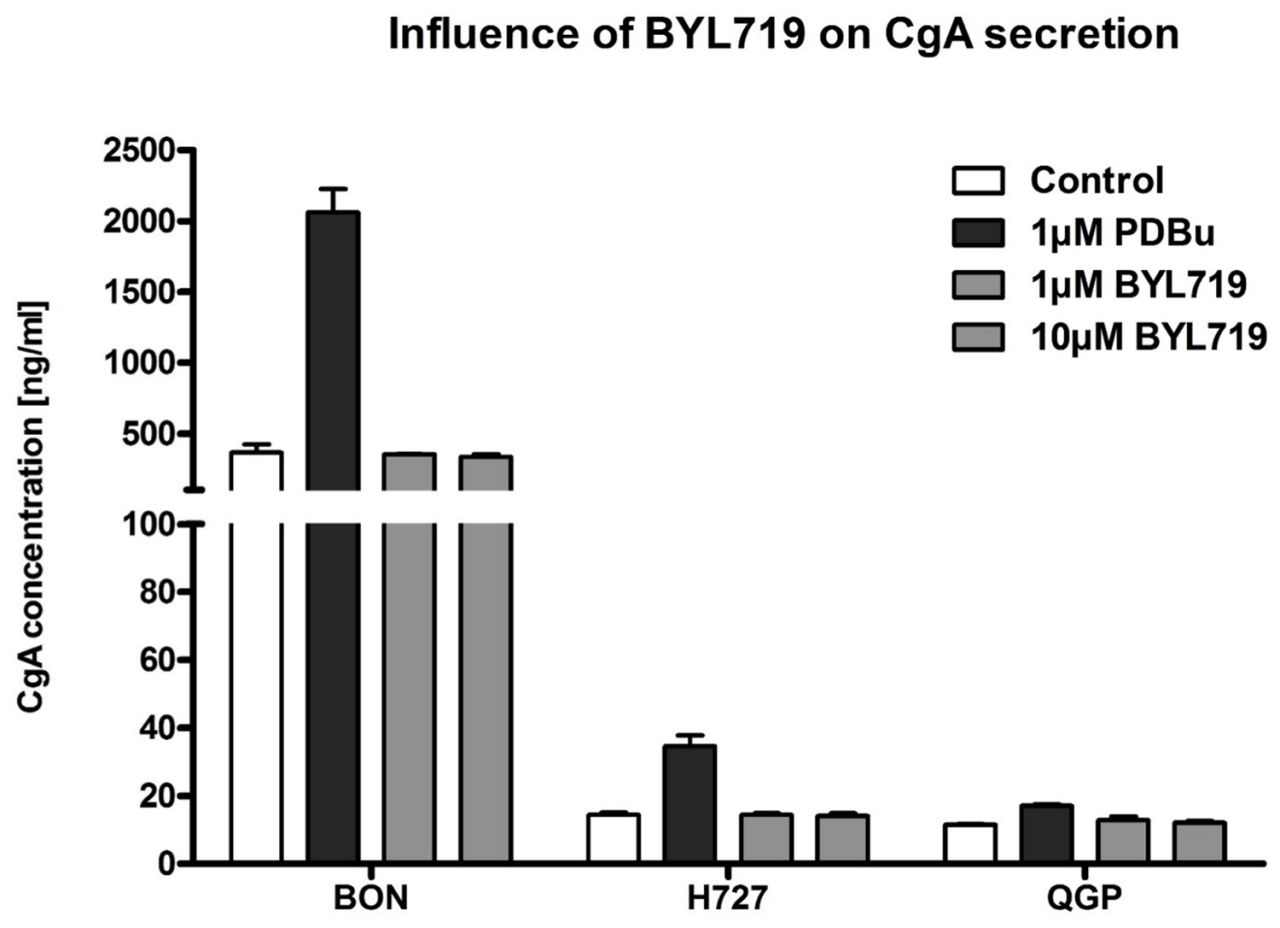
CgA secretion of NET cells was not altered by BYL719 treatment. BON-1, H727 and QGP-1 cells were treated with vehicle, PDBu (positive control) or BYL719 (1 μM, 10 μM) for 24 h; the supernatant was collected for CgA measurements (two independent experiments with three replicates per data point).

To evaluate the influence of BYL719-induced cell differentiation and signaling on CgA secretion in neuroendocrine tumor cells, BON-1, H727 and QGP-1 cells were analyzed for their release of CgA when treated with either vehicle, the known PKC activator and secretagogue phorbol-12,13-dibutyrate (PDBu), or two concentrations of BYL719. As Fig 6 demonstrates, PDBu at a concentration of 1 μM was able to increase the concentration of secreted CgA in BON-1 cells from the basal value (366 ± 58 U/ml) about 5-fold (2061 ± 166 U/ml). PDBu also raised CgA levels in the medium of H727 (about 2-fold) and QGP-1 cells (about 1.5-fold). In contrast, BYL719 did not alter the concentration of CgA in either BON-1, H727 or QGP-1 cell supernatants at either 1 or 10 μM concentrations (Fig 6).

### Synergistic effect of BYL719 plus everolimus in QGP-1 cells

**Fig 7:**
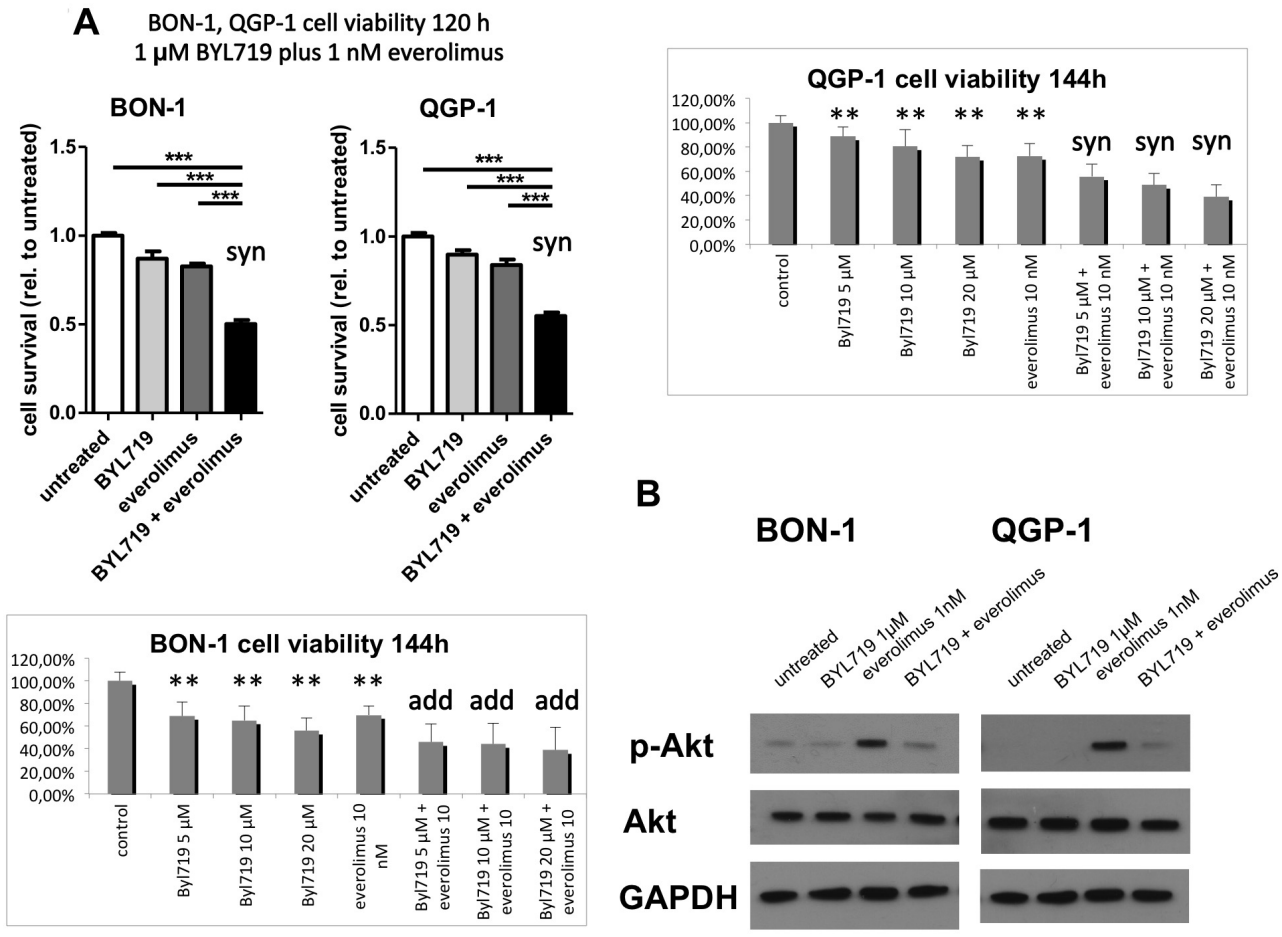
A: The combination therapy of BYL719 plus everolimus was synergistic in QGP-1 cells at all tested doses (1–20 μM BYL719 plus 1–10 nM everolimus) and in BON-1 cells at low doses (1 μM BYL719 plus 1 nM everolimus) (p≤0.01), and additive in BON-1 cells at higher doses. MTS cell viability assay data are shown as mean ± SD of 4–6 replicates per data point from two independent experiments, referred to the untreated control; syn: synergistic (p≤0.01); add: additive; **, *** highly significant, compared to the control (** p≤0.01, *** p≤0.001); B: Co-treatment of BON-1 and QGP-1 cells with BYL719 and everolimus prevented everolimus-induced AKT activation. Cells have been treated with 5 μM BYL719, 5 nM everolimus or the combination of both for 48h and subjected to western blot analysis of pAKT^Ser473^, AKT and GAPDH. Representative Western Blot of three replicates.

Given earlier reports on synergistic effects of combination therapies with PI3K and mTOR inhibitors, we evaluated the potential of co-treatment of BYL719 and everolimus in our NET cell line models *in vitro*. In QGP-1 cells—showing the lowest sensitivity to BYL719 alone—a synergistic inhibitory effect of 1–20 μM BYL719 plus 1–10 nM everolimus on cell viability was found (p≤0.01) (Fig 7A).

In BON-1 cells, there was also a synergistic effect at low doses of 1 μM BYL719 plus 1 nM everolimus, and an additive effect of both treatments at higher doses (Fig 7A), whereas H727 cells did not respond to everolimus and no synergistic or additive effect was observed in the latter (data not shown). As described earlier, Western blot analysis showed a strong activation of AKT after treatment with everolimus alone, which was almost completely prevented by addition of a low dose of 5 μM BYL719 in BON-1 and QGP-1 cells (Fig 7B).

**Fig 8:**
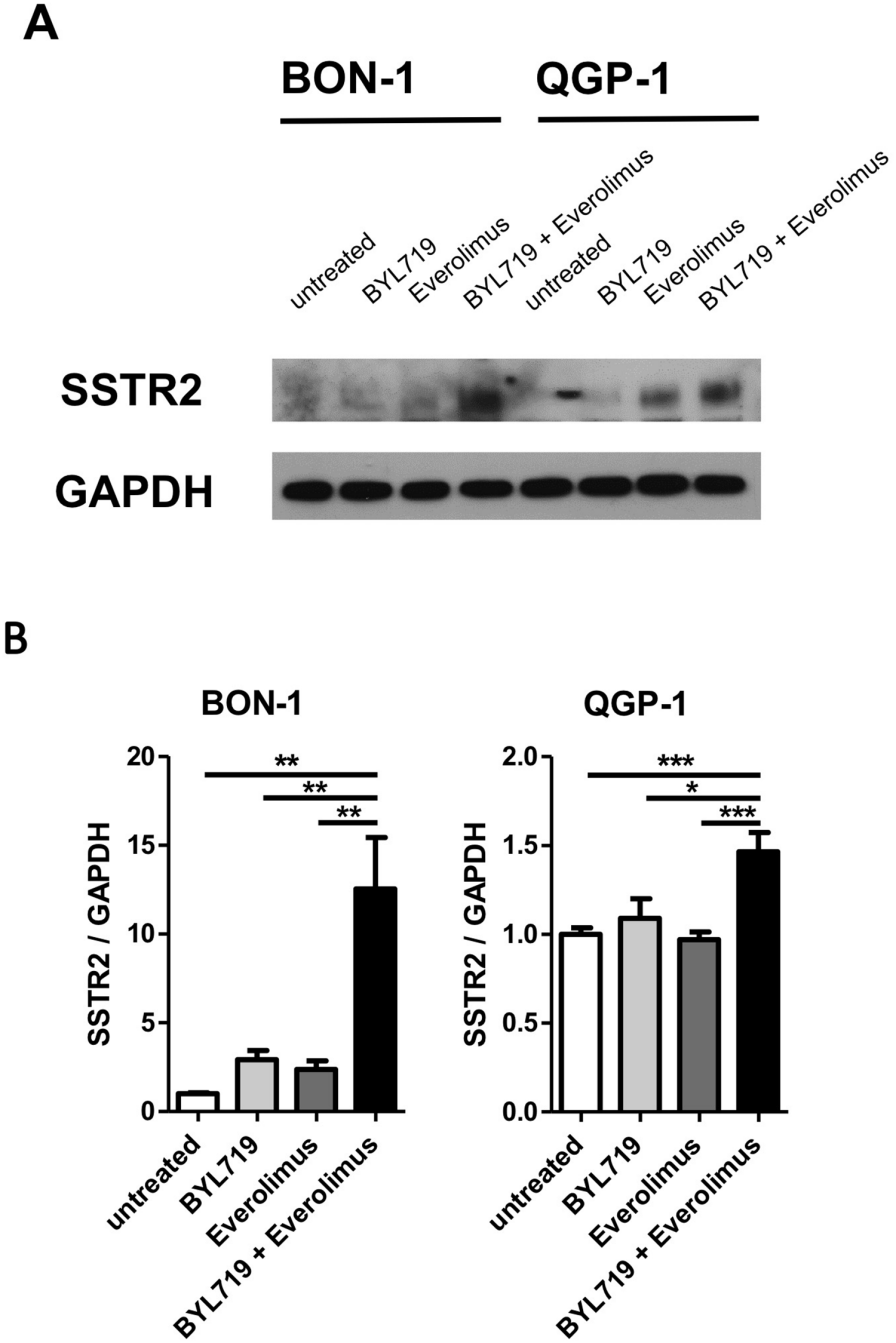
The combination therapy with BYL719 plus everolimus strongly induced SSTR2 expression in BON-1 and QGP-1 cells. Cells have been treated with 5 μM BYL719, 5 nM everolimus, or the combination of both for 48h (A) or with 1 μM BYL719, 1 nM everolimus or the combination of both for 96h (B). SSTR2 expression was analyzed by Western Blot (A) and qPCR (B). Representative western blot of two replicates; qPCR results are summarized from three independent experiments of three replicates. * p<0.05; ** p≤0.01; *** p≤0.001.

Moreover, 1 μM BYL719 plus 1 nM everolimus significantly more strongly induced *SSTR2* transcription compared to each drug separately in BON-1 (12.5-fold, CI 5.8–19.2) and QGP-1 cells (1.5-fold, CI 1.2–1.7) (Fig 8).

It should be noted that this synergism already occurred at doses (1 μM for BYL719 and 1 nM for everolimus), which do not influence cell viability as monotherapies.

### Comparison of BYL719 versus the panPI3K inhibitor BKM120 in BON-1 cells

To prove the involvement of other PI3K subunit isoforms apart from PI3Kα in NET signaling, we compared the efficacy of the selective PI3Kα inhibitor BYL719 to the efficacy of the panPI3K inhibitor BKM120 in the most sensitive cell line BON-1 cells as our model, and found a much stronger inhibitory effect of BKM120 at low doses, compared to BYL719 (IC_20_ BKM120 at 72h 0.5 μM vs. IC_20_ BYL719 at 72 h 6.9 μM; IC_50_ BKM120 at 72 h 1.2 μM vs. IC_50_ BYL719 at 72 h not reached) (S28 Fig).

## Discussion

### Cell viability, apoptosis and cell cycle

BYL719 dose-dependently reduced NET cell viability and colony formation with highest sensitivity for BON-1, followed by H727 cells and with the lowest sensitivity for QGP-1 cells. Our data are consistent with a previous study showing higher sensitivity of BON-1 cells to BYL719 compared to QGP-1 cells [18]. Interestingly, colony formation was already suppressed at low doses (IC_50_ 1.3 μM in BON-1 and 1.8 μM in QGP-1 cells). The colony formation assay tests every cell in the population for its ability to undergo “unlimited” division and determines reproductive cell death after treatment with cytotoxic agents [50]. Therefore, colony formation may also reflect interactions between tumor cells and its microenvironment and might even better correlate with tumor growth *in vivo* than cell viability assays [51–53].

In all three NET cell lines investigated, BYL719 induced apoptosis associated with significantly increased caspase 3 and. cleaved PARP. The highest sensitivity to BYL719 correlated with strongest BYL719-induced apoptosis in BON-1 cells, followed by H727 cells and then QGP-1 cells.

Moreover, BYL719 induced cell cycle arrest in the G_0_/G_1_-phase in all three cell lines. This was consistent with a significant up-regulation of p27 in all cell lines investigated. The strongest BYL719-mediated increase of p27 after 24 h treatment was found in BON-1 cells. A positive correlation of loss of p27 and aggressiveness as well as increased cell cycle progression of gastroenteropancreatic NETs has previously been described [54].

After 24 h of BYL719 incubation, there was a significant decrease of activated DNA-damage checkpoint marker Chk1 in all three cell lines. Interestingly, inhibition of Chk1 has shown promising anti-cancer effects *in vitro* in a variety of p53 deficient human tumor cell lines through induction of caspase-3/7 dependent apoptosis, and especially sensitized colon tumors to chemotherapy *in vivo* [55, 56]. This may be an interesting additional effect in poorly differentiated neuroendocrine carcinomas (NECs) which regularly bear p53 mutations [57]. The observed significant decrease of the cell cycle inhibitor p21 may be due to PI3K/AKT inhibition by BYL719, since PI3K/AKT signaling increases p21 [58, 59]. The role of p21 has been a controversal topic in the literature [60]. On the one hand, it is a cell cycle inhibitor leading to cell cycle arrest and anti-proliferative signals. On the other hand, there is also evidence that p21 may inhibit apoptosis, promote tumor cell growth and cooperate with Chk1 to prevent apoptosis during DNA replication fork stress [59–63]. Therefore, the observed decrease of p21 due to partial PI3K/AKT inhibition together with a Chk1 inhibition may also have pro-apoptotic/anti-tumor effects.

### Signaling pathways

#### Inhibition of PI3K/AKT/mTORC1/2 signaling

In all three cell lines, mTORC2-induced PI3K/AKT^*Ser473*^ signaling was significantly and partially, but not completely, inhibited by BYL719 treatment, and the inhibition was stronger in BON-1 and QGP-1, compared to H727 cells. Similar data were found for PI3K/PDK1/AKT^Thr308^ signaling with significant partial inhibition in BON-1 and QGP-1 cells, but even activation in H727 cells after 24 h of treatment with 10 μM BYL719. This suggests that, surprisingly, potent PI3K/AKT inhibition may not be crucial for BYL719 efficacy in NET cells. This indicates potential non-selective effects of BYL719 independent of the p110α subunit and the involvement of alternative signaling pathways, as discussed in detail below. Consistently, we could show only slight inhibitory effects of a low dose of 1 μM BYL719 alone on cell viability, also arguing for non-selective effects of BYL719 at higher doses.

Moreover, this indicates the potential importance of alternative PI3Kp110 catalytic subunit isoforms in NET signaling. PI3K110α (the catalytic subunit of class I PI3K) has been considered as the only relevant catalytic subunit in the context of cancer associated mutations [64] and has shown relevant anti-tumor potential in pancreatic NETs *in vitro* and *in vivo* [18, 65]. However, the other variants of the p110 catalytic subunit—β or δ—may be of importance and promote sustained activity of PI3K/AKT^Ser473^ and PI3K/AKT^Thr308^ despite PI3K110α inhibition. The δ isoform of the p110 catalytic subunit of PI3K has already shown anti-tumor potential in leukemia or in anaplastic thyroid carcinoma [66, 67]. The theory of potential involvement of alternative PI3K subunit isoforms in NET signaling was supported by a much stronger inhibitory effect of the panPI3K inhibitor BKM120, compared to BYL719, on BON-1 cell viability (IC_20_ BKM120 at 72h 0.5 μM vs. IC_20_ BYL719 at 72h 6.9 μM; IC_50_ BKM120 at 72h 1.2 μM vs. IC_50_ BYL719 at 72h not reached).

Interestingly, while we confirmed previous findings of a partial mTORC1 inhibition at 10 μM BYL719 in pancreatic NET cells [18], we noted that H727 cells showed more pronounced mTORC1 inhibition accompanied by higher PI3K/AKT activity in response to BYL719 treatment. Thus, our data might argue for a direct effect of BYL719 on mTORC1. In this context, PI3K knock-down experiments would seem to be a reasonable procedure to prove a direct impact of BYL719 on mTORC1 signaling and will be subject of further studies. However, with our current study we were not able to confirm a direct effect of BYL719 on mTORC1. BON-1 and QGP-1 cells behave very similarly with respect to the impact of BYL719 on PI3K/AKT/mTORC1 signaling although they showed different sensitivities. BON-1 cells showed lower inhibition on PI3K/AKT signaling, compared to QGP-1 cells, and the lowest inhibition of mTORC1 of all three cell lines, however, the highest sensitivity to BYL719 of all three cell lines. Hence, the extent of PI3K/AKT/mTORC1 inhibition did not seem to correlate with sensitivity to BYL719 in the different NET cell lines. This implicates another mechanism of action different from PI3K/AKT/mTORC1 signaling responsible for the different cell line sensitivities.

#### Inhibition of GSK3 in BON-1 and H727 cells, but not in QGP-1 cells at 24 h treatment

The most evident difference between the three cell lines which might correlate with different cell line sensitivities was found in GSK3 signaling. GSK3 is usually phosphorylated and inhibited by the growth factor induced PI3K/AKT signaling pathway [46–48]. Hence, partial PI3K/AKT inhibition would be expected to lead to dephosphorylation/activation of GSK3. However, unexpectedly, despite partial PI3K/AKT inhibition, BYL719 treatment led to phosphorylation/inhibition of GSK3 in BON-1 and H727 cells with a significant and strongest inhibition, especially of GSK3β in the most sensitive BON-1 cell line. Hence, our study suggests an impact of BYL719 on GSK3 not directly linked to PI3K/AKT signaling. In contrast to GSK3 inhibition in BON-1 and H727 cells, there was *no phosphorylation/inhibition*, *but a modest activation* of GSK3 in the more resistant QGP-1 cells after BYL719 treatment.

The role of GSK3β in cancer has been discussed and remains controversial [46, 68]. Since GSK3β induces phosphorylation and degradation of its downstream targets, and some GSK3β substrates are key proteins for promoting cell survival, such as β-Catenin, Cyclin D1, eIF2B and MYC [46, 68, 69], it was initially considered as a tumor suppressor. However, in contrast, a tumor-*promoting* role of GSK3β has been reported in leukemia, glioblastoma, non-small cell lung cancer, BON-1, insulinoma and H727 cells as well as medullary thyroid carcinoma cells [70–75]. Accordingly, GSK3β inhibition has already shown anti-tumor potential with anti-proliferative and pro-apoptotic effects in neuroblastoma, BON-1, H727 and medullary thyroid carcinoma cells being accompanied by reduced expression of β-catenin and neuroendocrine tumor markers [70, 71, 76, 77]. GSK3β inhibition also displayed anti-proliferative effects in other tumor entities, including leukemia, non-small cell lung cancer, melanoma, prostate, pancreatic, colon and renal cell carcinoma [73, 75, 78–83]. Several studies have found that inhibition of GSK3β led to apoptosis through inhibition of NFκB, mitotic catastrophe in apoptosis-resistant cells, and cycle arrest due to the increase of CDK inhibitors including p27 *in vitro* and *in vivo* [46, 71, 84–88]. This is consistent with our findings of *strongest* GSK3 inhibition being associated with *highest sensitivity* to BYL719 and strongest induction of apoptosis in BON-1, compared to H727 and QGP-1 cells, after BYL719 treatment. One potential explanation for this effect of BYL719 on GSK3 would be IGF1 receptor activation in all three cell lines to a different extent in response to BYL719 treatment. This theory, however, could not be supported by our data, as discussed in detail below. A more detailed analysis of signaling effects is warranted in novel primary cell lines and primary tumor cell cultures to understand differences in signaling pathway alterations in NETs.

#### Compensatory activation of IGF1R

Svejda *et al*. have already reported a growth factor mediated feedback loop in NET cells after mTORC1 inhibition [19], as we observed with BYL719 in this study: we found a strong activation of IGF1R in all cell lines, which was already seen at low doses of 1 μM BYL719 arguing for a specific effect of PI3Kα inhibition on IGF1R signaling. In case of inappropriate strong mTORC1 activation, a negative feed-back effect on IGFR signaling has previously been described [89]. Therefore, the observed activation of IGF1 receptor might be due to PI3K/mTORC1 inhibition and might be a potential compensatory mechanism of the tumor cells. IGF1R activation seems to play an essential role in cancer cell proliferation of different tumor cell entities including BON-1 cells [90–94].

On the other hand, IGF1R is known to phosphorylate/inhibit GSK3 [47]. Therefore, we have tested a potential dependence of GSK3 phosphorylation on IGF1R signaling in our NET cell lines through co-treatment of BYL719 with the specific IGF1R inhibitor NVP-AEW541. Surprisingly, in BON-1 and H727 cells co-treatment with the specific IGF1R inhibitor NVP-AEW541 even led to an enhanced (not reduced) GSK3 phosphorylation/inhibition, compared to each drug separately, being associated with strongest IGF1R activation after combination treatment in BON-1 and H727 cells; in QGP-1 cells, absent GSK3 phosphorylation/inhibition, compared to the control, was accompanied by IGF1R inhibition after combination treatment. NVP-AWE541 alone seemed to lead to a compensatory increase of total IGF1R expression, potentially to compensate for the IGF1R inhibition, and thereby rendering cells more sensitive to BYL719-induced IGF1R activation.

However, the low baseline phosphorylation/activity of the IGF1R in BON1 and H727 cells might not have been sufficient to show the effects of specific IGF1R inhibition by NVP-AEW541; moreover, another group stimulated their cells with IGF1 to demonstrate the dependence of GSK3 phosphorylation on IGF1R activity in medulloblastoma cells [49],. Therefore, we modified our experimental setting by using IGF1 stimulation. IGF1 stimulation led to strong basal IGF1R phosphorylation/activity in all three cell lines, which could then be clearly inhibited by NVP-AEW541 in all three cell lines. BYL719 alone led to strong IGF1R phosphorylation/activation. However, despite clear IGF1R inhibition after combination treatment with NVP-AEW541 plus BYL719 in all three cell lines, the phosphorylation/inhibition of GSK3 was even stronger (not blocked) after the combination treatment in H727 cells and not changed in BON-1 and QGP-1 cells compared to single BYL719 treatment. Therefore, according to these data, blockade of the IGF1R did not attenuate BYL719-induced GSK3 phosphorylation. Due to optimizing our experimental design by IGF1 stimulation according to a previously published experimental design [49], we have refuted our initial hypothesis of the dependence of BYL719-induced GSK3 phosphorylation on IGF1R activation in BON1 and H727 cells. The mechanism through which BYL719 leads to GSK3 inhibition, thus still needs to be assessed and will be the subject of further studies. Essentially, the IGF1R does not appear to mediate the effect of Byl719 on the phosphorylation of GSK3.

### Induction of cell re-differentiation in BON-1 and H727 cells, but not in QGP-1 cells

BYL719 induced neuroendocrine differentiation in BON-1 and H727 cells as indicated by increased expression of CgA (*CHGA-)*, *SSTR1-* and *SSTR2*-mRNA; *SSTR2* mRNA expression was already significantly induced at a low dose of 1 μM BYL719 arguing for a specific effect of PI3Kα inhibition on *SSTR2*-mRNA induction; in contrast, in QGP-1 cells, *SSTR1* transcripts were reduced, and *CHGA* and *SSTR2* were unaffected.

Although *CHGA*-mRNA was found to be upregulated by BYL719, the compound did not modulate CgA release from any of the cell lines studied. It was important to verify this, as Li et al. have reported that the p110 catalytic subunit of PI3K negatively regulated neurotensin secretion in BON-1 cells and that inhibition of p110 induced neurotensin release from these cells [95]. Likewise, inhibition of the PI3K/PDK1/AKT pathway has been shown to upregulate insulin secretion from pancreatic beta cells [96]. Potentiation of hormone hypersecretion in NET patients would be considered an unwanted side effect that could limit the application of this therapeutic principle. However, none of the three cell lines tested showed an increase of CgA secretion under BYL719 treatment. In clinical practice, CgA is a marker for NETs in terms of progression and changes in response to therapy, but has low sensitivity for diagnosis, especially when the tumor burden is low. It tends to be higher in more differentiated tumors, but there is considerable variability and in general it is not universally useful in determining the degree of tumor differentiation [97].

In contrast, SSTR1/2 expression seems to be a more suitable marker for cell differentiation. In general, almost 100% of low (G1) and intermediate grade (G2) NETs show SSTR expression, but high grade NETs and neuroendocrine carcinomas (NECs) (G3) show lesser expression. Hence, in less differentiated tumors SSTR expression is less frequent and SSTR density is lower. Moreover, SSTR expression is less frequently seen in pancreatic than in gastrointestinal tumors, as reviewed in [98]. Furthermore, SSTR2 expression positively correlated with better prognosis In NET patients [99, 100]. Therefore, re-differentiation of tumor cells with an increase of SSTR2 expression, as we observed after BYL719 and after BYL719/everolimus combination treatment (discussed below) *in vitro*, might lead to better differentiated tumors and improve the prognosis of NET patients if transferable to humans. Moreover, the increased SSTR1/2 expression upon BYL719 exposure could potentially be exploited for co-treatments with somatostatin analogs. It also seems an interesting option to increase SSTR2 mediated tracer uptake for imaging studies and peptide radioreceptor ligand therapy (PRRT). Interestingly, SSTR5 seems to be differentially regulated compared to SSTR1 and SSTR2.

GSK3 inhibition might promote neuronal and pancreatic NET cell differentiation, as described previously [101, 102]. Therefore, pronounced BYL719-induced inhibition of GSK3 in BON-1 and H727 cells may contribute to enhanced tumor cell differentiation; accordingly, there was no inhibition, but even a slight activation of GSK3 in QGP-1 cells after 24 h BYL719 treatment associated with decreased cell differentiation.

Potentially, the BYL719-induced cell differentiation in BON-1 and H727 cells reflects re-differentiation of tumor cells. A re-differentiation of thyroid carcinoma cells upon growth arrest has been described previously [103]. Indeed, it has recently been reported, that treatment of BON-1 cells with HDAC inhibitors can potently induce SSTR2 expression coinciding with a profound proliferative arrest of the tumor cells [104].

### Synergistic effect of BYL719 plus everolimus in QGP-1 cells

Although there was just a slight effect of a low dose of 1 μM BYL719 alone on BON-1 and no relevant effect on QGP-1 cell viability, there was a synergistic effect of 1 μM BYL719 in combination with the mTORC1-inhibitor everolimus (1 nM) in both cell lines. This may be explained by additional everolimus-mediated mTORC1 inhibition rendering cells more sensitive to BYL719, as previously reported for breast cancer cells [38], and BYL719-mediated prevention of everolimus-induced AKT activation [16–18], as now shown in our cells. Moreover, in another study on BON-1 cells (submitted data) we have shown a much stronger phosphorylation/inhibition of GSK3 after 72 h BYL719/everolimus combination treatment (by 2507%), compared to each drug separately (by 195% and 298%, respectively). This again supports the concept of combining targeted therapies in NETs, as previously shown to be effective: Combined inhibition of mTORC1/2, PI3K/mTORC1/2, PI3K/mTORC1/2/ERK or EGFR/PI3K/mTORC1 has shown promising anti-tumor potential in NETs *in vitro* [18, 28, 42, 45, 105]. Of note implementing combination therapies could dramatically reduce the doses of single agent treatment, as we observed synergistic effects already at subtherapeutic doses in monotherapy. Moreover, since these synergistic effects already occurred at low doses of both drugs, the synergism might be a specific effect of simultaneous inhibition of the PI3Kα subunit by BYL719 and of mTORC1 by everolimus. Nevertheless, harmful side effects of combined treatments should be evaluated in animal studies.

Furthermore, we found that the synergism of 1 μM BYL719 plus 1 nM everolimus was associated with induction of cell re-differentiation and a significant increase of SSTR2 expression in QGP-1 and BON-1 cells, compared to each drug separately, potentially sensitizing cells to somatostatin analogs.

No synergistic or additive effect of the BYL719/everolimus combination was found in H727 cells. This is consistent with our finding of lower PI3K/AKT^*Ser473*^ inhibition and even activation of PI3K/AKT^Thr308^ associated with stronger mTORC1 inhibition by BYL719 alone in H727 cells.

## Conclusions

The selective PI3Kα inhibitor BYL719 significantly reduced cell viability and colony formation in all NET cell lines investigated, with the highest sensitivity in BON-1, followed by H727 cells, and a much lower sensitivity in QGP-1 cells. The highest sensitivity of BON-1, followed by H727 cells, might be due to stronger BYL719-induced apoptosis as a potential consequence of GSK3 inhibition and tumor cell re-differentiation with significant SSTR1/2 up-regulation, in contrast to lower apoptosis induction, absent GSK3 inhibition and absent re-differentiation in QGP-1 cells [71, 72, 85, 101, 106, 107]. Thus, BYL719 may sensitize neuroendocrine tumor cells to therapy with somatostatin analogs or PRRT, depending on the specific cell line. Moreover, BYL719/everolimus combination treatment showed synergistic effects in BON-1 and the more resistant QGP-1 cells and might help to overcome everolimus or BYL719 resistance through simultaneous AKT/mTORC1 inhibition. The BYL719/everolimus combination also enhanced cell differentiation with significant SSTR2 up-regulation in BON-1 and QGP-1 cells, compared to each drug separately.

In summary, our suggested hypothesis and our data indicate that BYL719 might be an interesting novel therapeutic option for NETs, especially in combination with the approved and promising somatostatin analogs or everolimus, which merits clinical therapeutic consideration. However, our data also confirm the importance of individualized targeted therapies depending on tumor entity.

## Supporting information

S1 Fig Uncropped western blots. (TIF)

S2 Fig Uncropped western blots. (TIF)

S3 Fig Uncropped western blots. (TIF)

S4 Fig Uncropped western blots. (TIF)

S5 Fig Uncropped western blots. (TIF)

S6 Fig Uncropped western blots. (TIF)

S7 Fig Uncropped western blots. (TIF)

S8 Fig Uncropped western blots. (TIF)

S9 Fig Uncropped western blots. (TIF)

S10 Fig Uncropped western blots. (TIF)

S11 Fig Uncropped western blots. (TIF)

S12 Fig Uncropped western blots. (TIF)

S13 Fig Uncropped western blots. (TIF)

S14 Fig Uncropped western blots. (TIF)

S15 Fig Uncropped western blots. (TIF)

S16 Fig Uncropped western blots. (TIF)

S17 Fig Uncropped western blots. (TIF)

S18 Fig Uncropped western blots. (TIF)

S19 Fig Uncropped western blots. (TIF)

S20 Fig Uncropped western blots. (TIF)

S21 Fig Uncropped western blots. (TIF)

S22 Fig Uncropped western blots. (TIF)

S23 Fig Uncropped western blots. (TIF)

S24 Fig Western blot 1 μM and higher concentrations of BYL719 pGSK3 and pIGF1R. (TIF)

S25 Fig Western blot 2.5 μM and higher concentrations of BYL719 pGSK3 and pIGF1R. (TIF)

S26 Fig Western blot NVP-AEW541 plus BYL719 without IGF1 stimulation. (TIF)

S27 Fig Western blot NVP-AEW541 plus BYL719 with IGF1 stimulation. (TIFF)

S28 Fig Comparison of the effects of BYL719 versus BKM120 on BON-1 cell viability. (TIF)

S29 Fig Uncropped western blots. (TIF)

S30 Fig Uncropped western blots. (TIF)

S1 Table Densitometry analysis of the performed western blots. Band intensities were quantified from at least 3 independent experiments for each cell line and protein, and are expressed as the mean percentage relative to the untreated control (100%). The means and standard deviations are reported as geometric means and geometric standard deviations of the relative increase, respectively. A geometric mean of "1.0" has to be interpreted as "equal to the control group" and for the geometric standard deviation “1.0” refers to “no variation“. Statistically significantly different results in comparison to the control are shown as p-values, considering p<0.05 as significant.
(XLSX)
