# Supplementary figures and images for: The selective PI3Kα inhibitor BYL719 as a novel therapeutic option for neuroendocrine tumors: Results from multiple cell line models
Source: PLoS One. 2017 Aug 11;12(8):e0182852. doi: 10.1371/journal.pone.0182852 (PMC5553670; doi:10.1371/journal.pone.0182852)

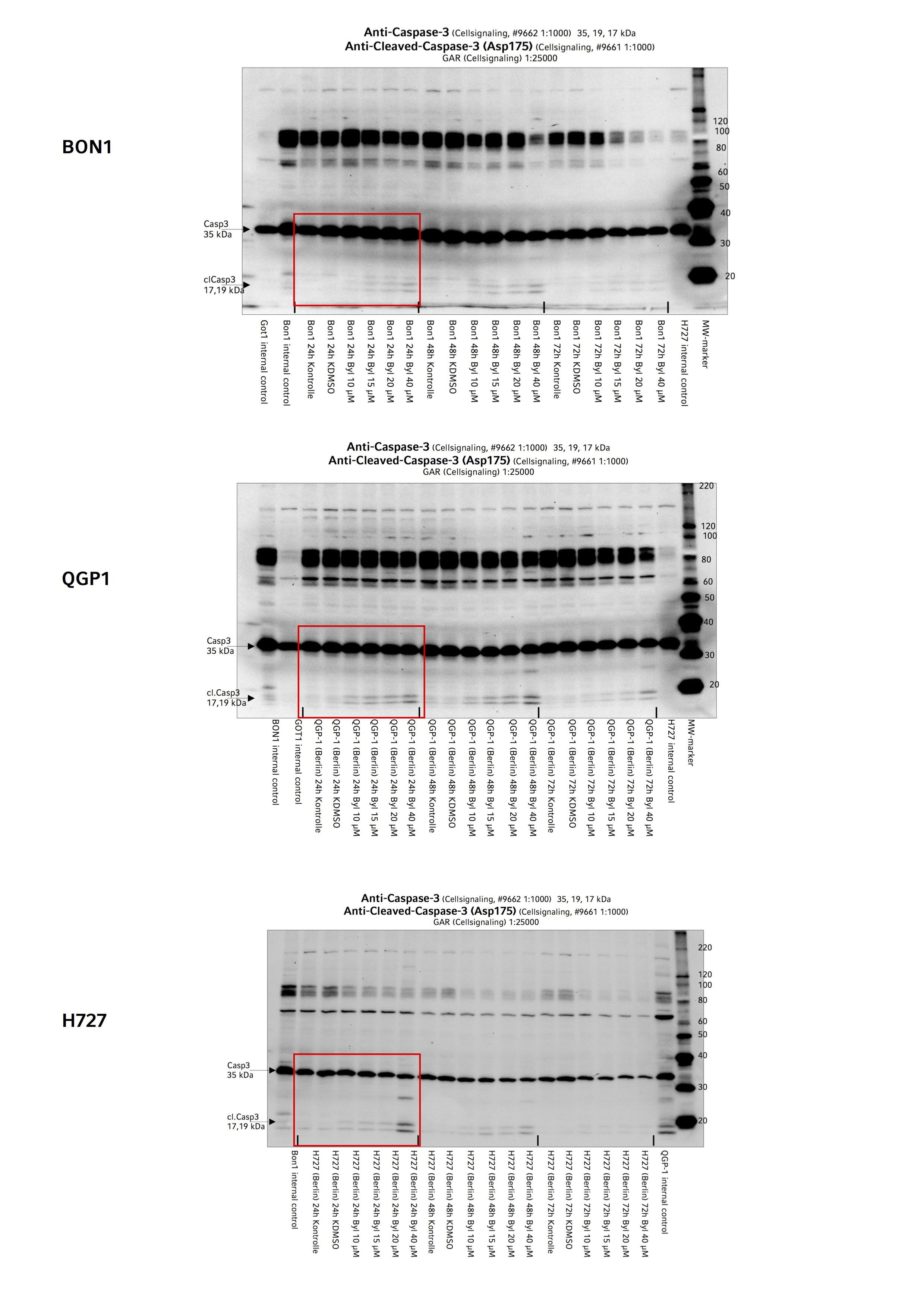

Supplement: S1 Fig — (TIF) [file pone.0182852.s001.tif]

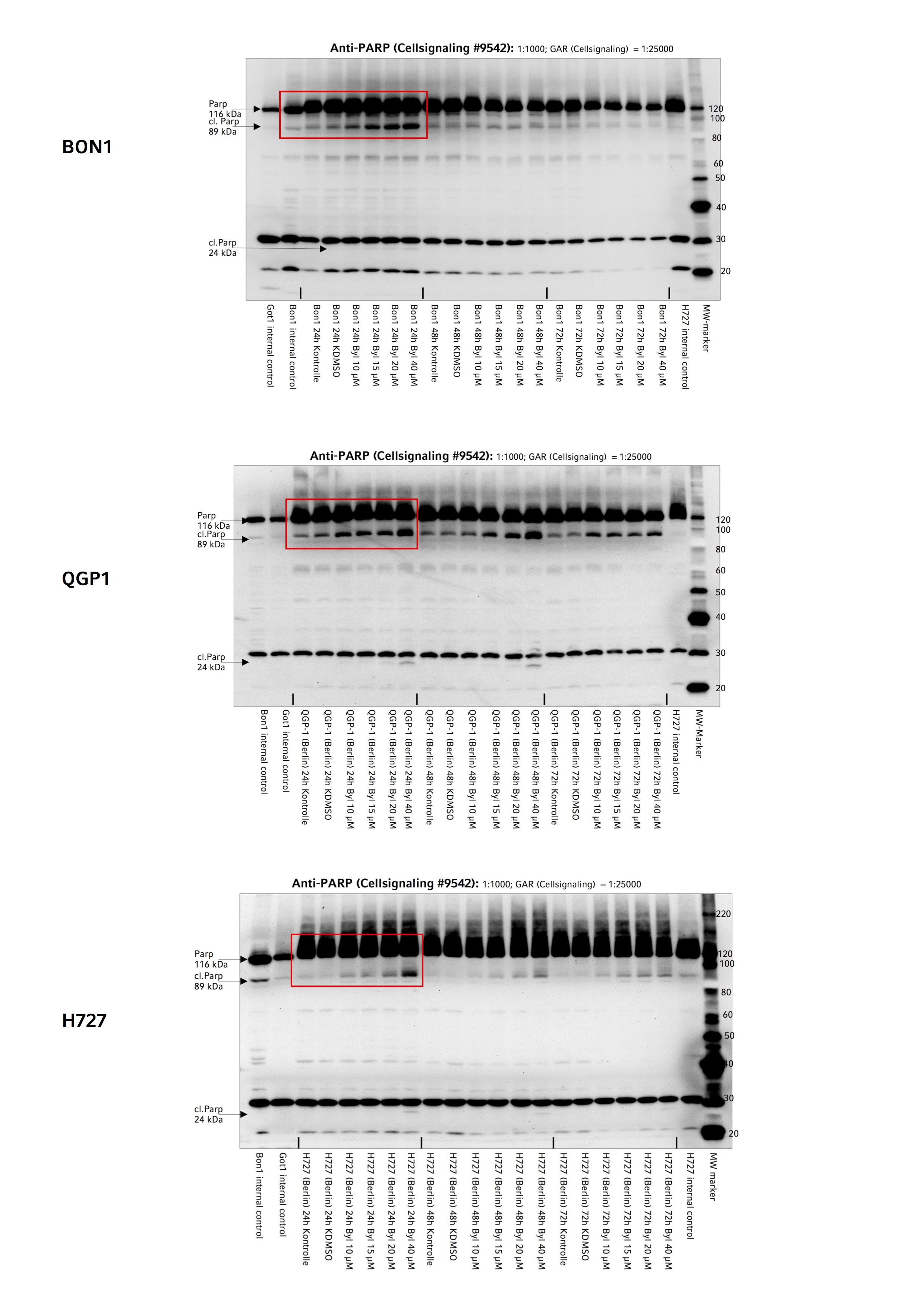

Supplement: S2 Fig — (TIF) [file pone.0182852.s002.tif]

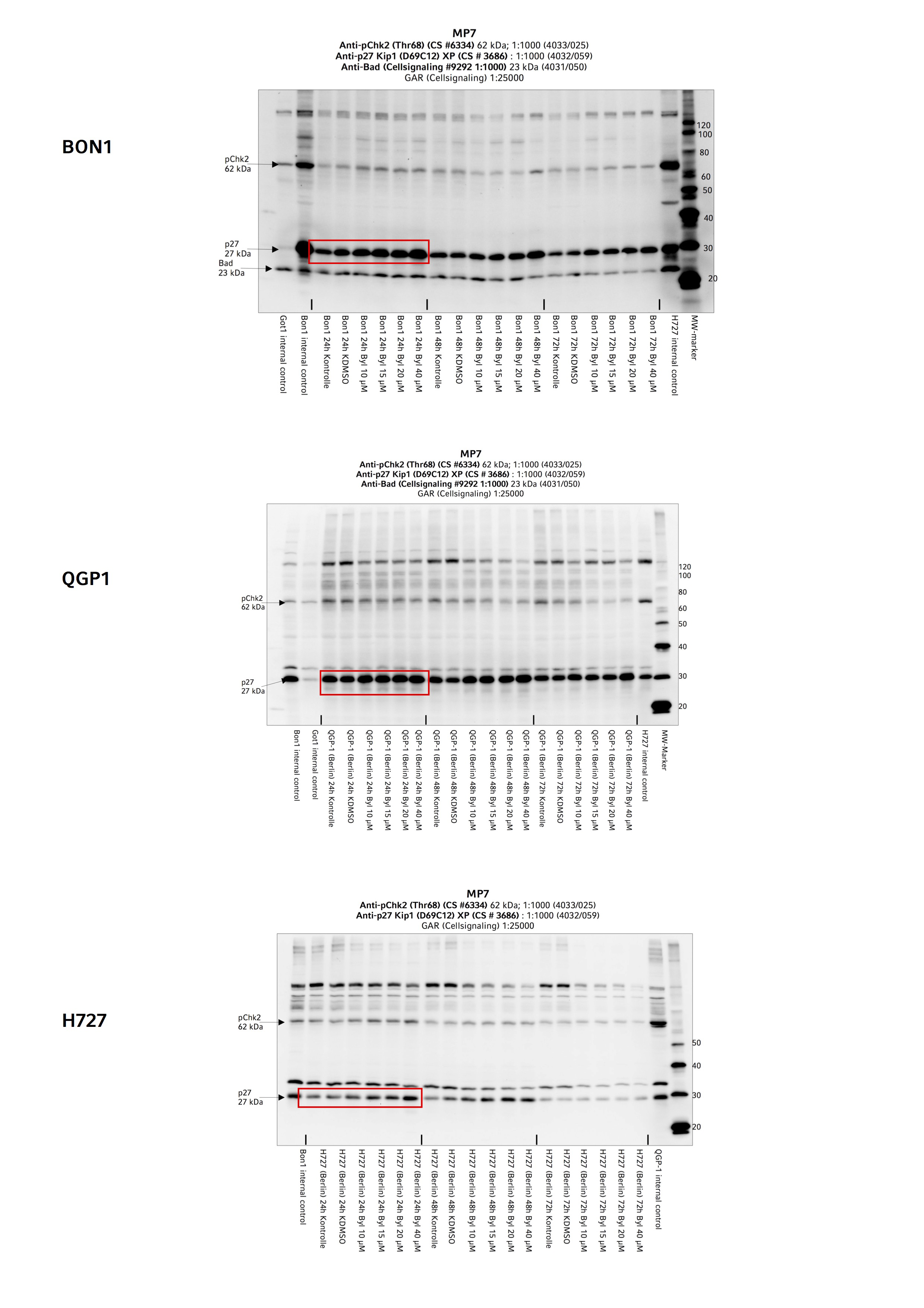

Supplement: S3 Fig — (TIF) [file pone.0182852.s003.tif]

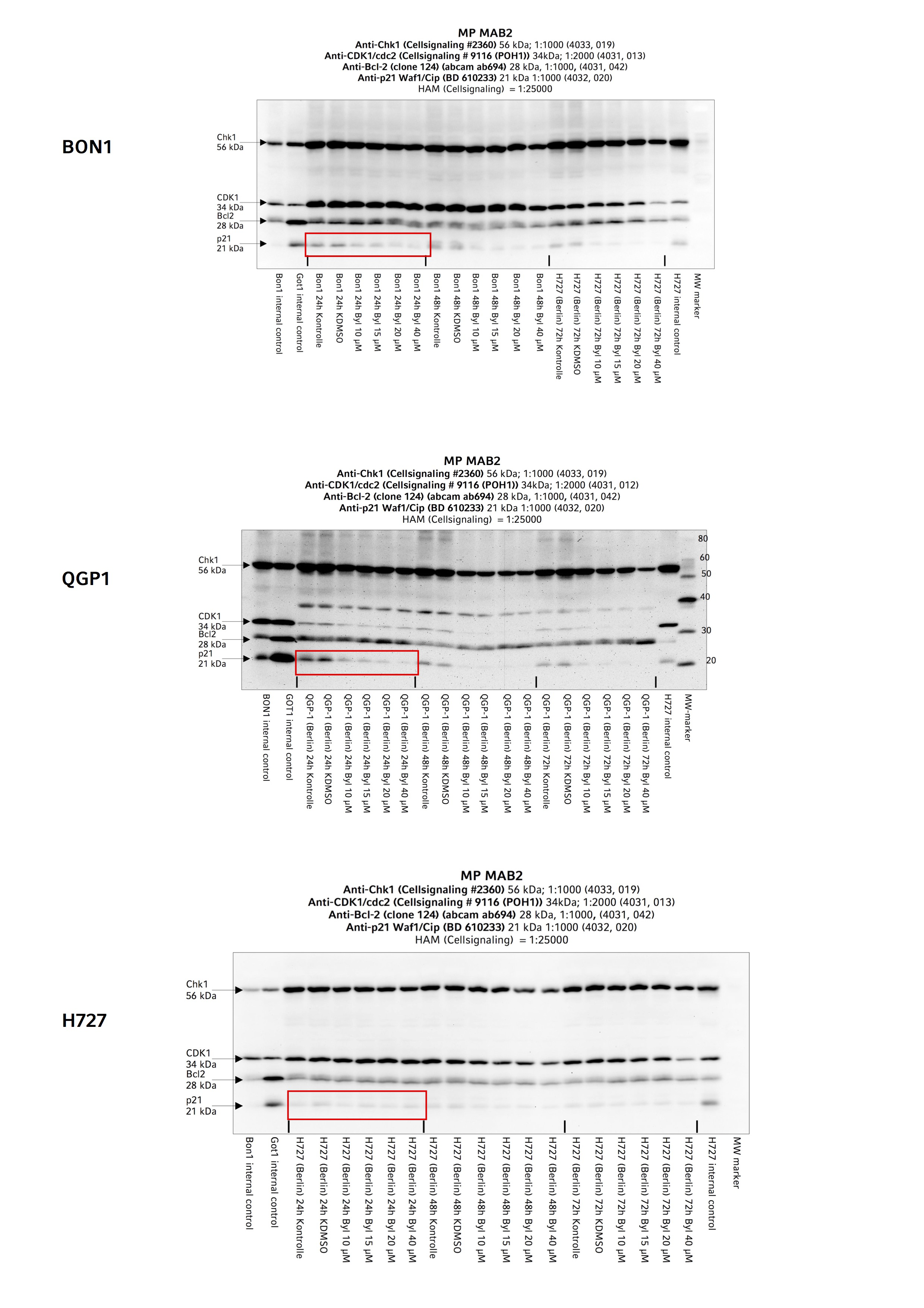

Supplement: S4 Fig — (TIF) [file pone.0182852.s004.tif]

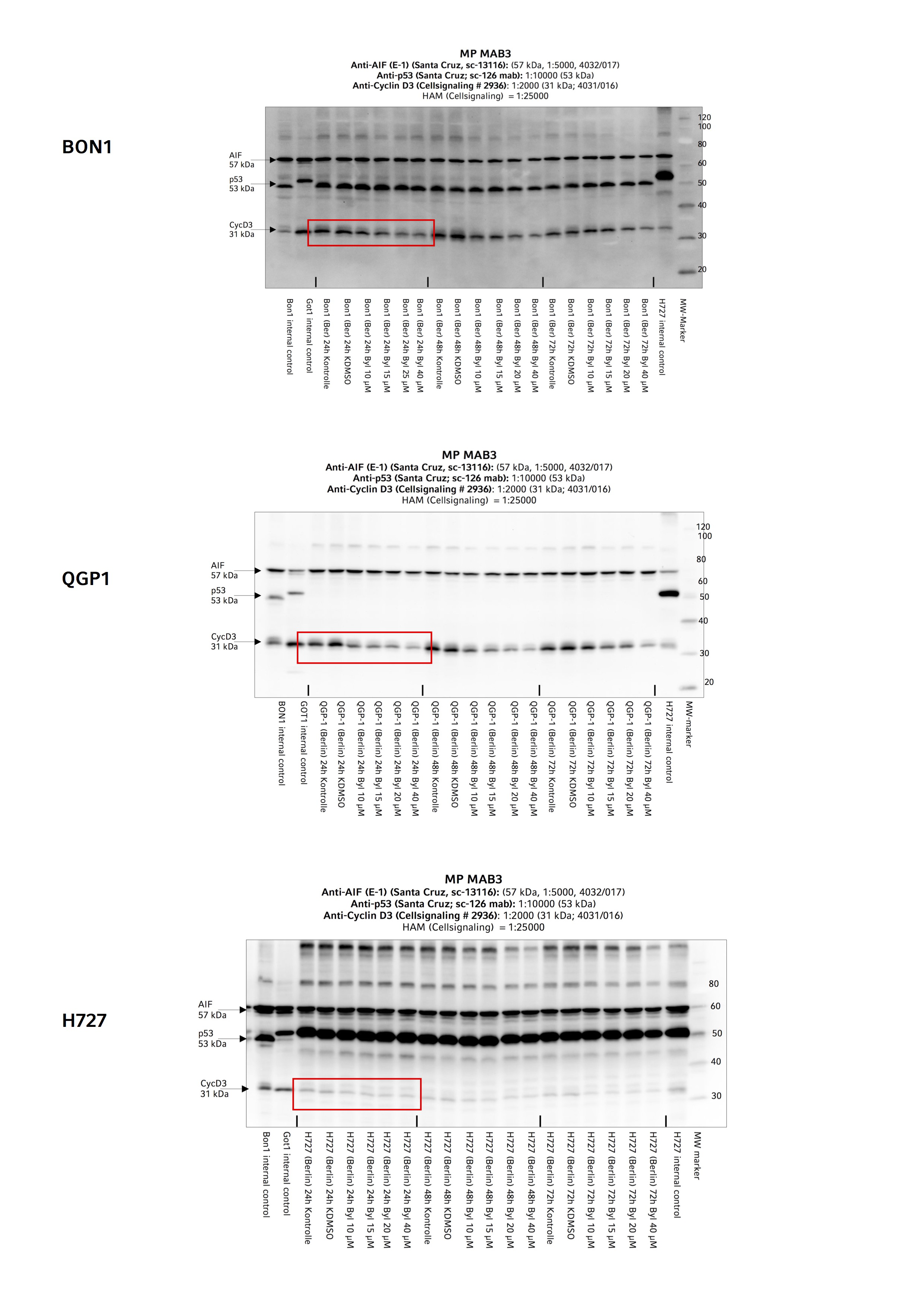

Supplement: S5 Fig — (TIF) [file pone.0182852.s005.tif]

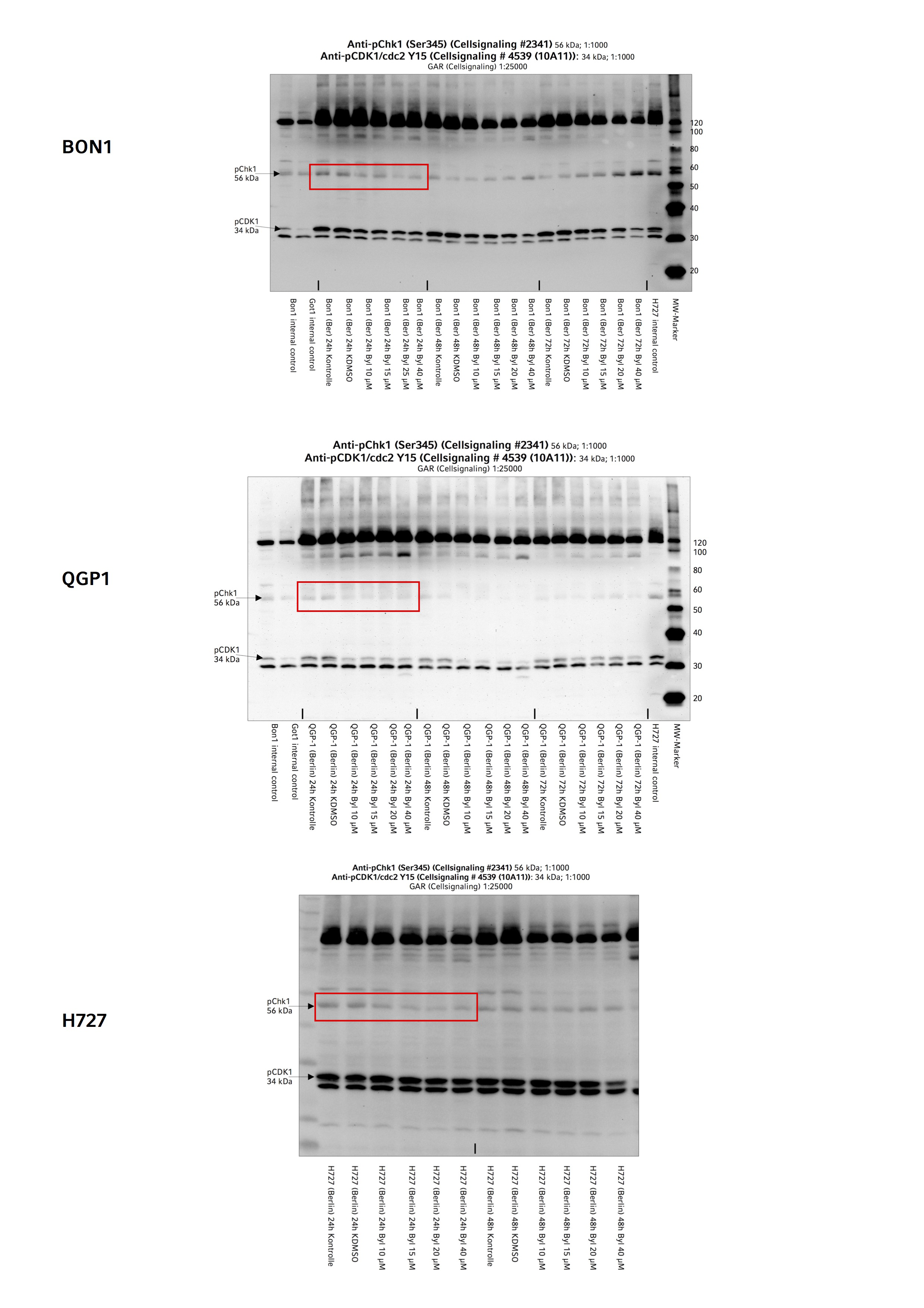

Supplement: S6 Fig — (TIF) [file pone.0182852.s006.tif]

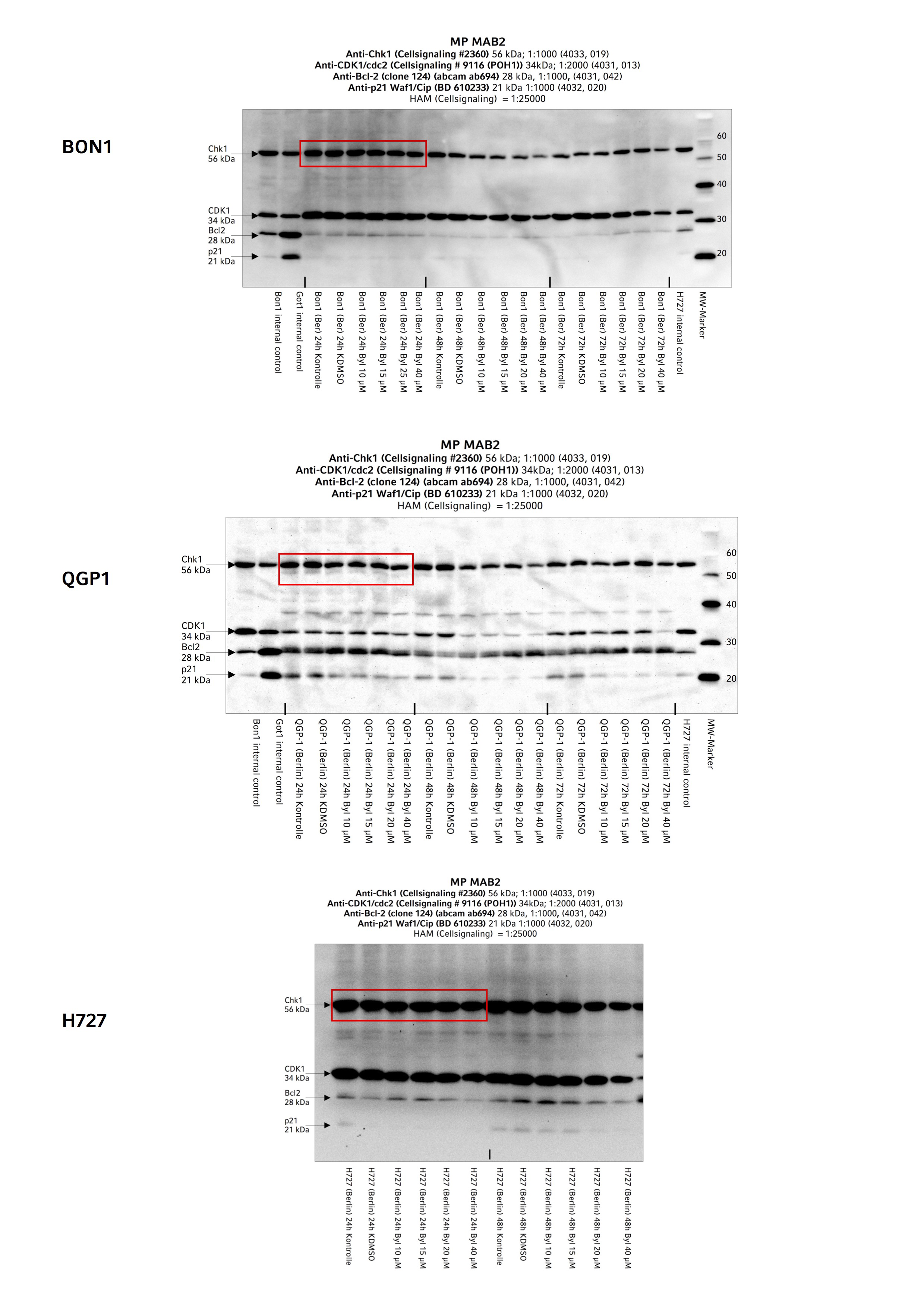

Supplement: S7 Fig — (TIF) [file pone.0182852.s007.tif]

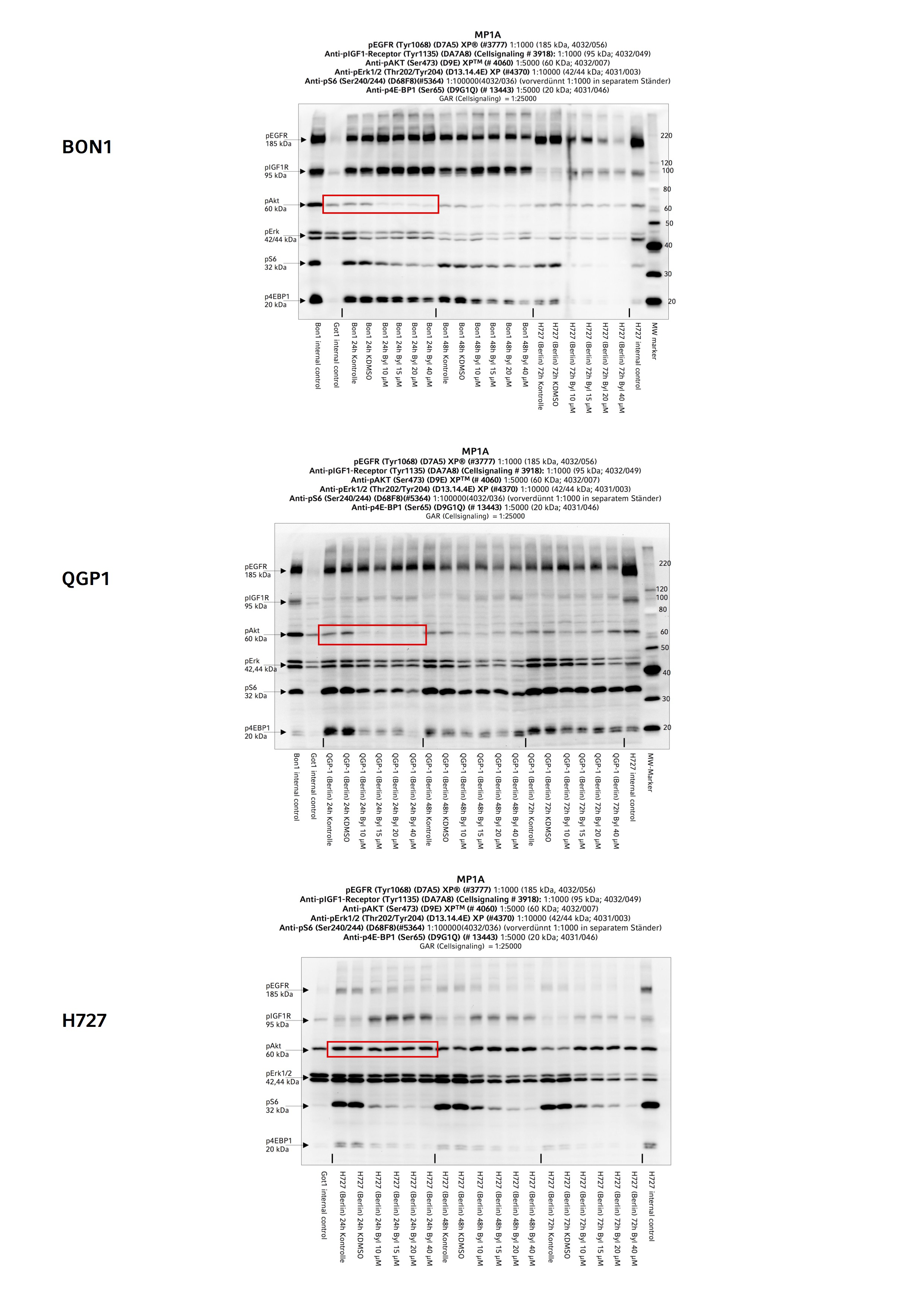

Supplement: S8 Fig — (TIF) [file pone.0182852.s008.tif]

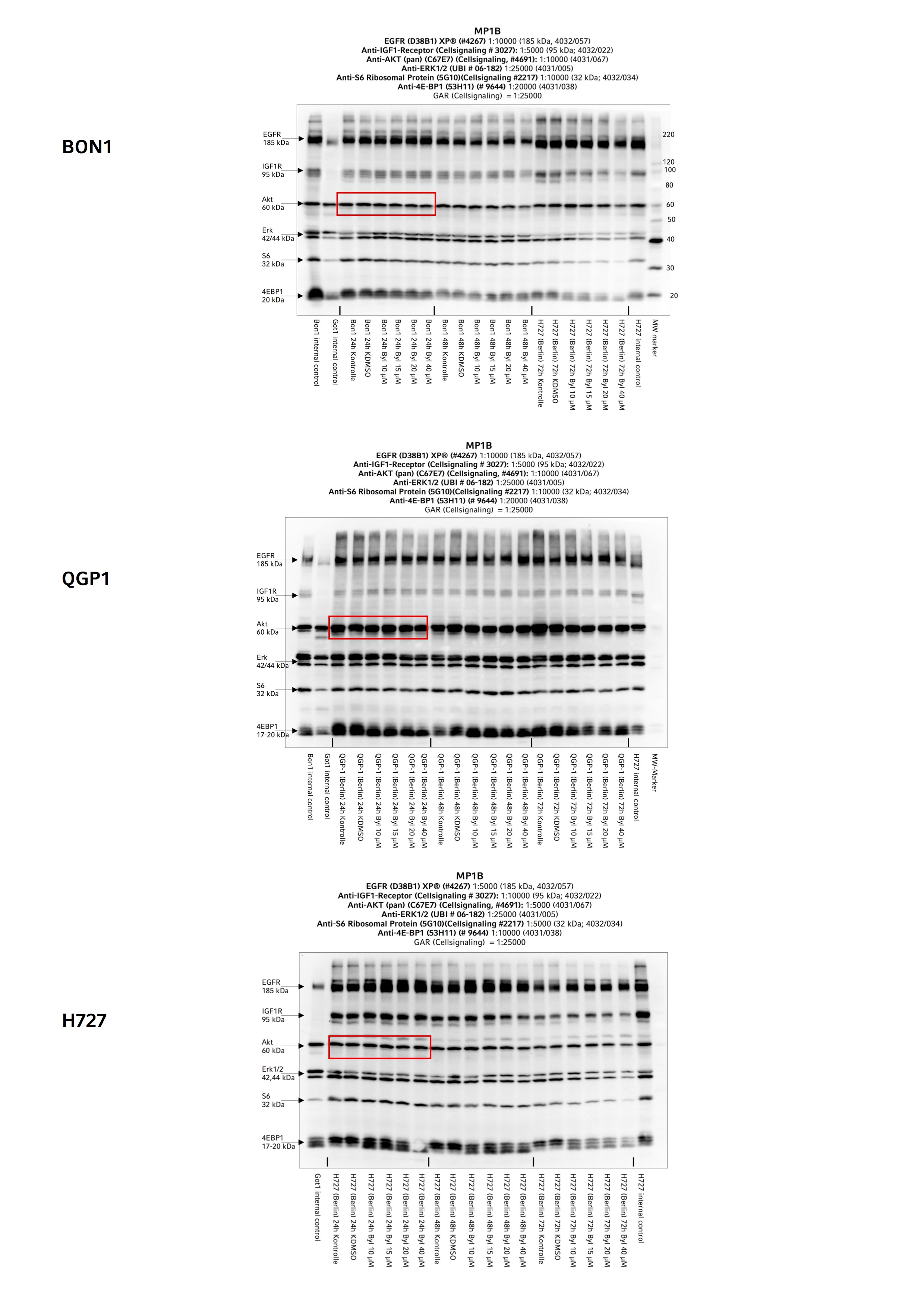

Supplement: S9 Fig — (TIF) [file pone.0182852.s009.tif]

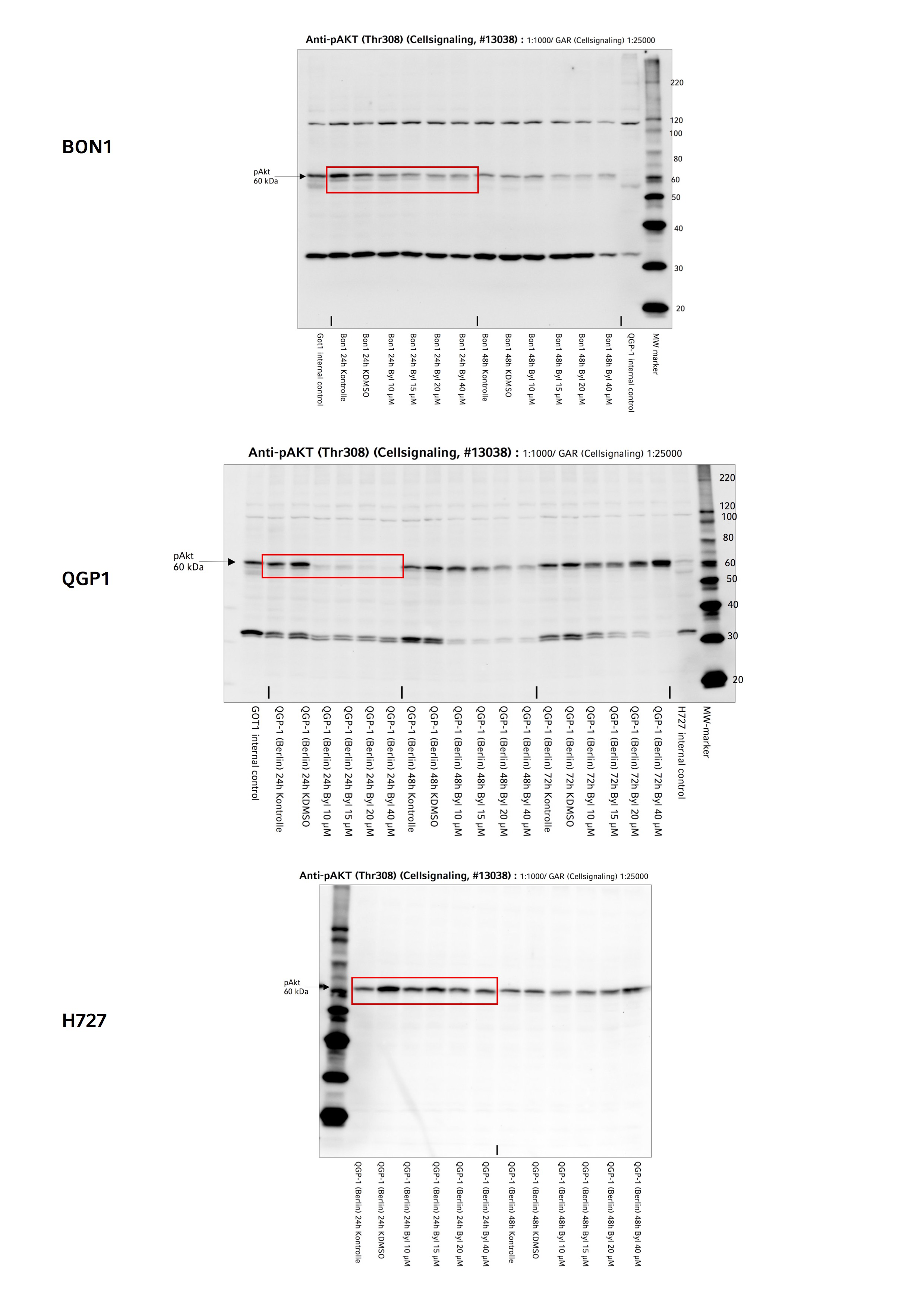

Supplement: S10 Fig — (TIF) [file pone.0182852.s010.tif]

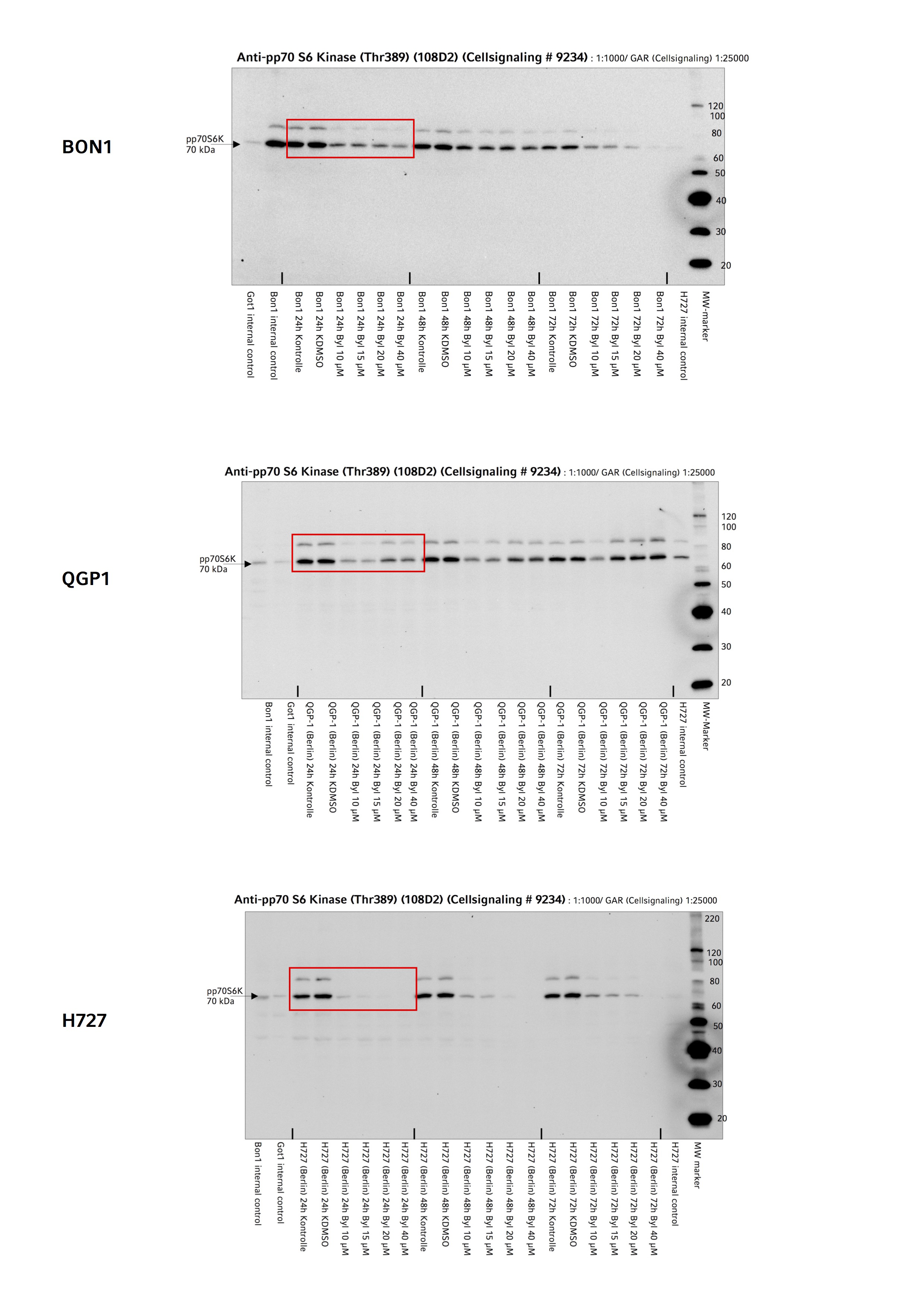

Supplement: S11 Fig — (TIF) [file pone.0182852.s011.tif]

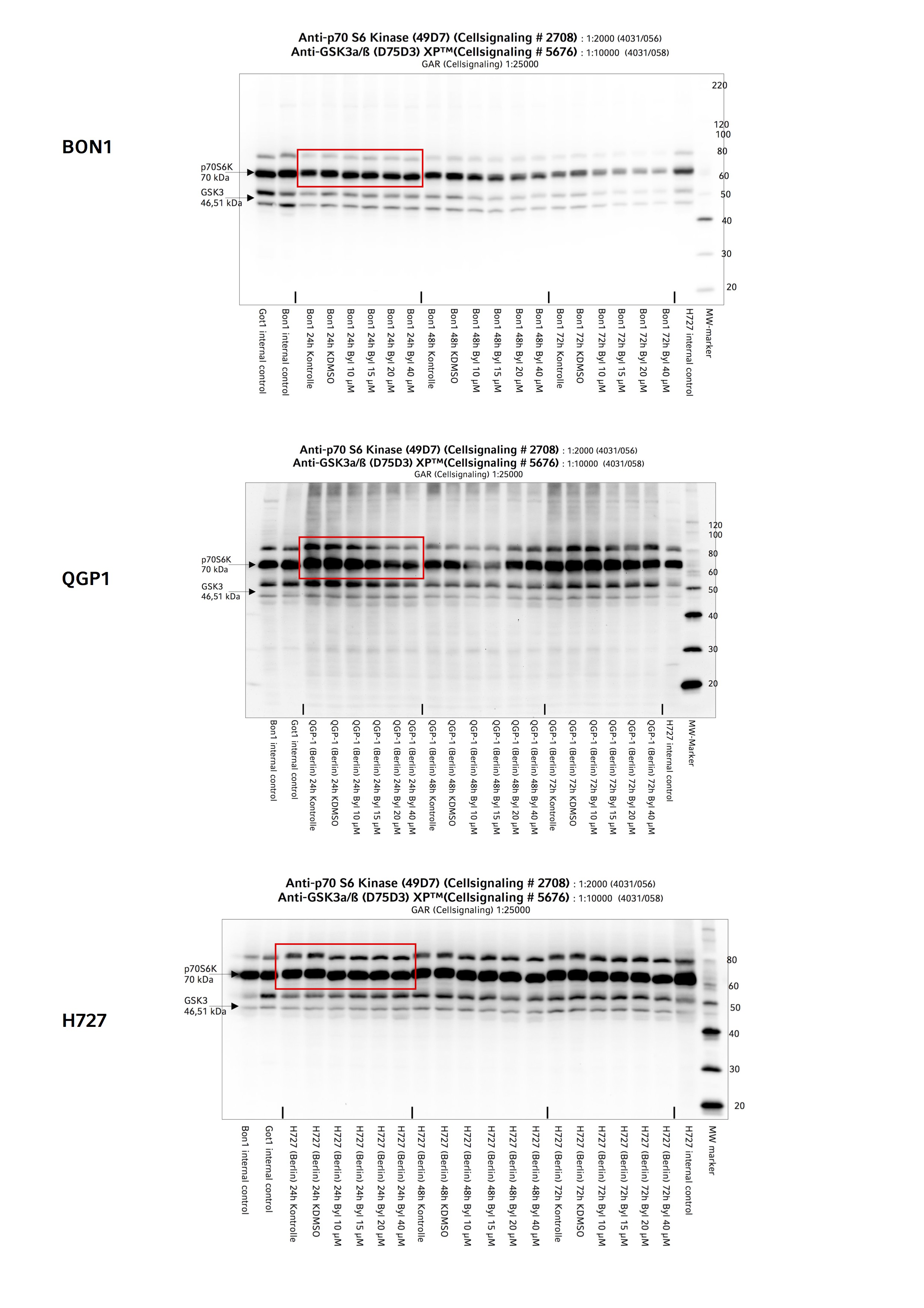

Supplement: S12 Fig — (TIF) [file pone.0182852.s012.tif]

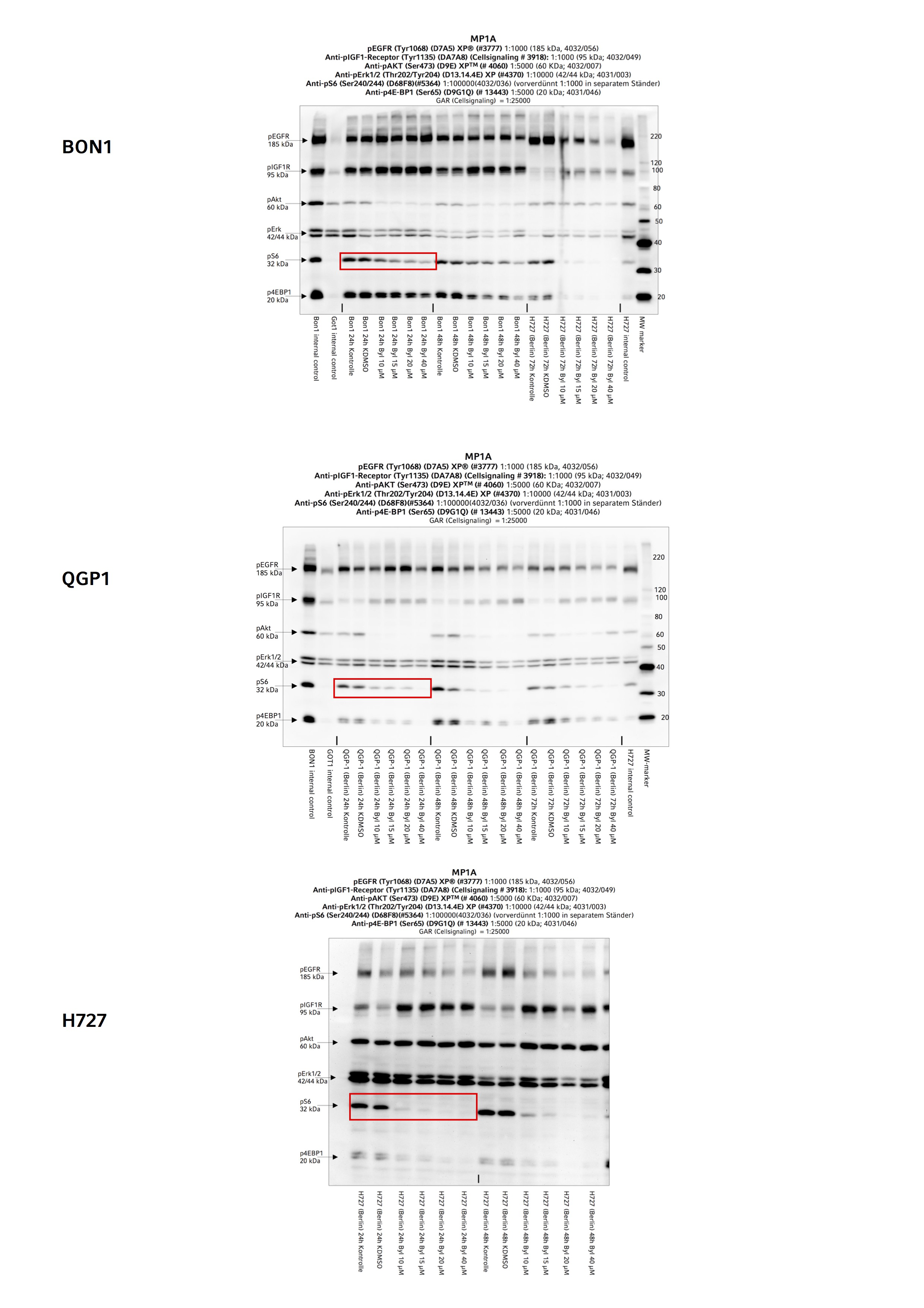

Supplement: S13 Fig — (TIF) [file pone.0182852.s013.tif]

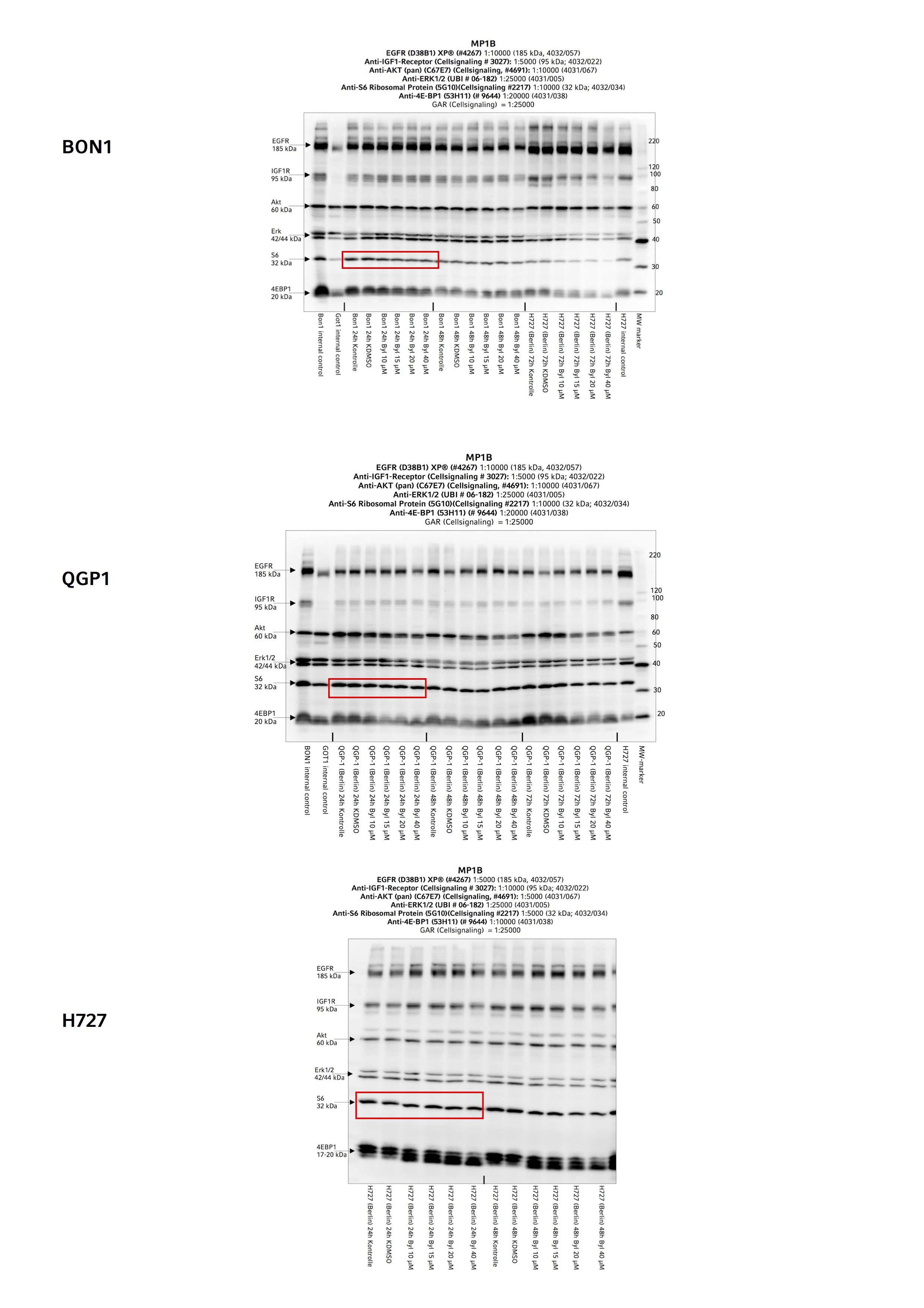

Supplement: S14 Fig — (TIF) [file pone.0182852.s014.tif]

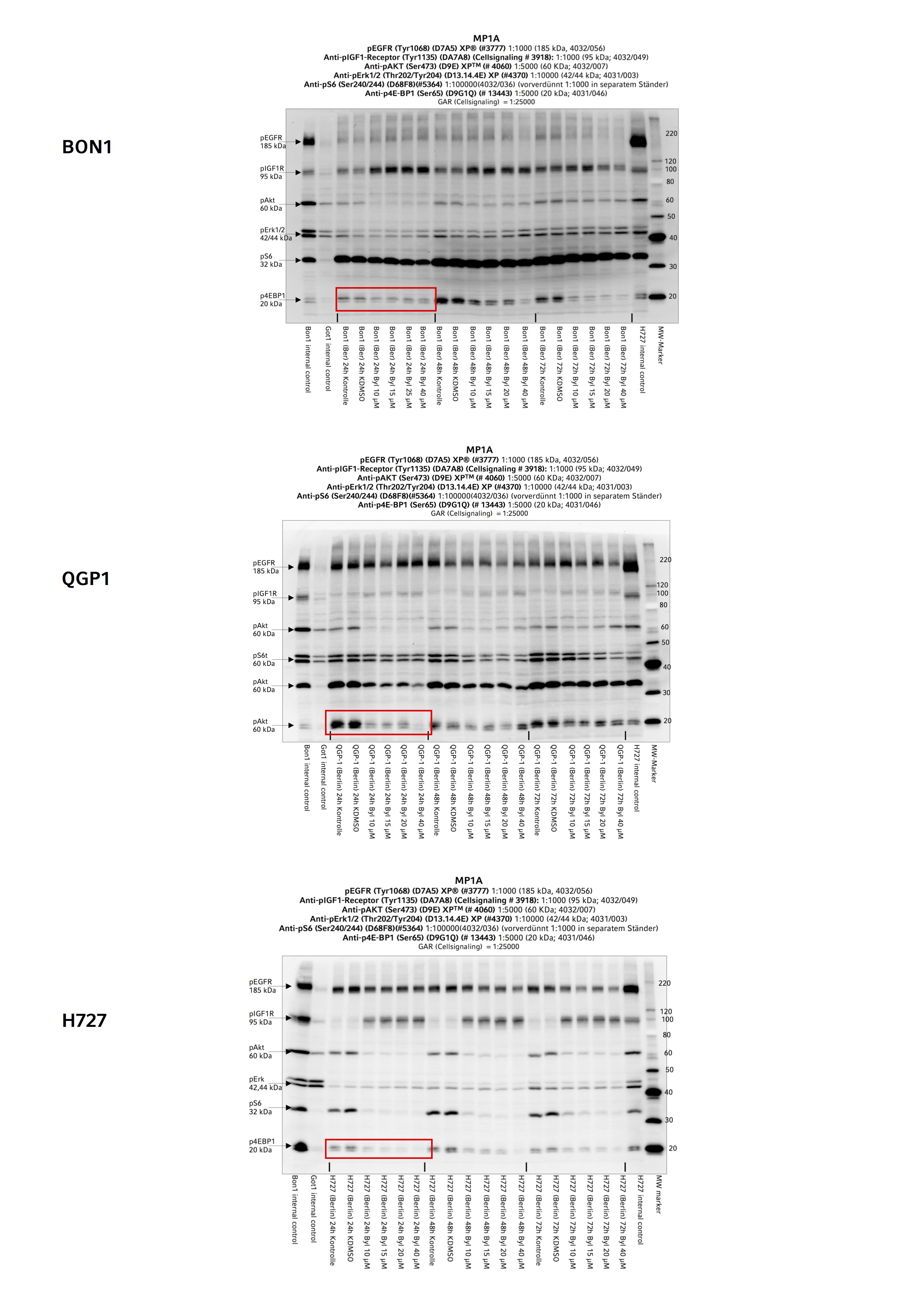

Supplement: S15 Fig — (TIF) [file pone.0182852.s015.tif]

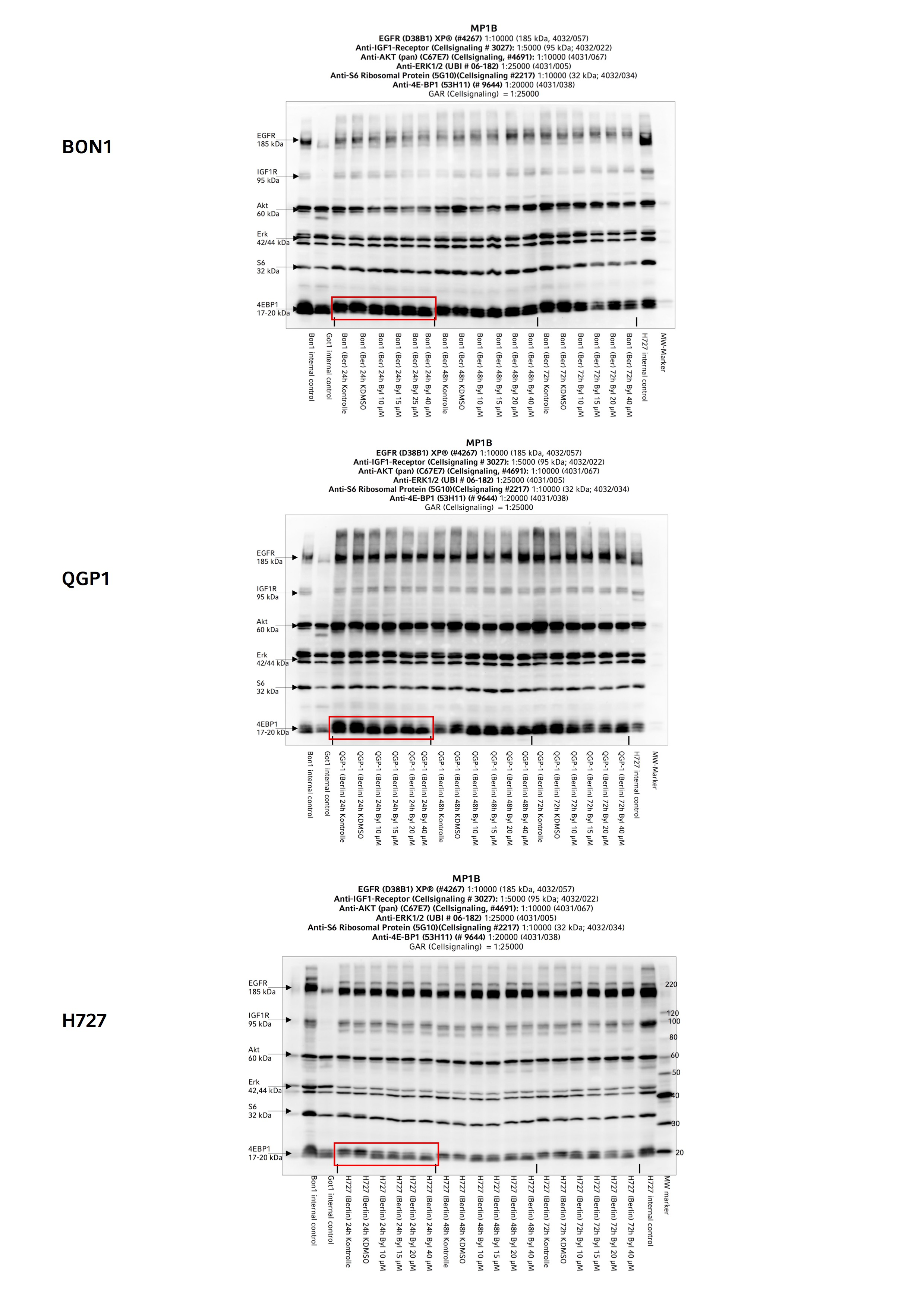

Supplement: S16 Fig — (TIF) [file pone.0182852.s016.tif]

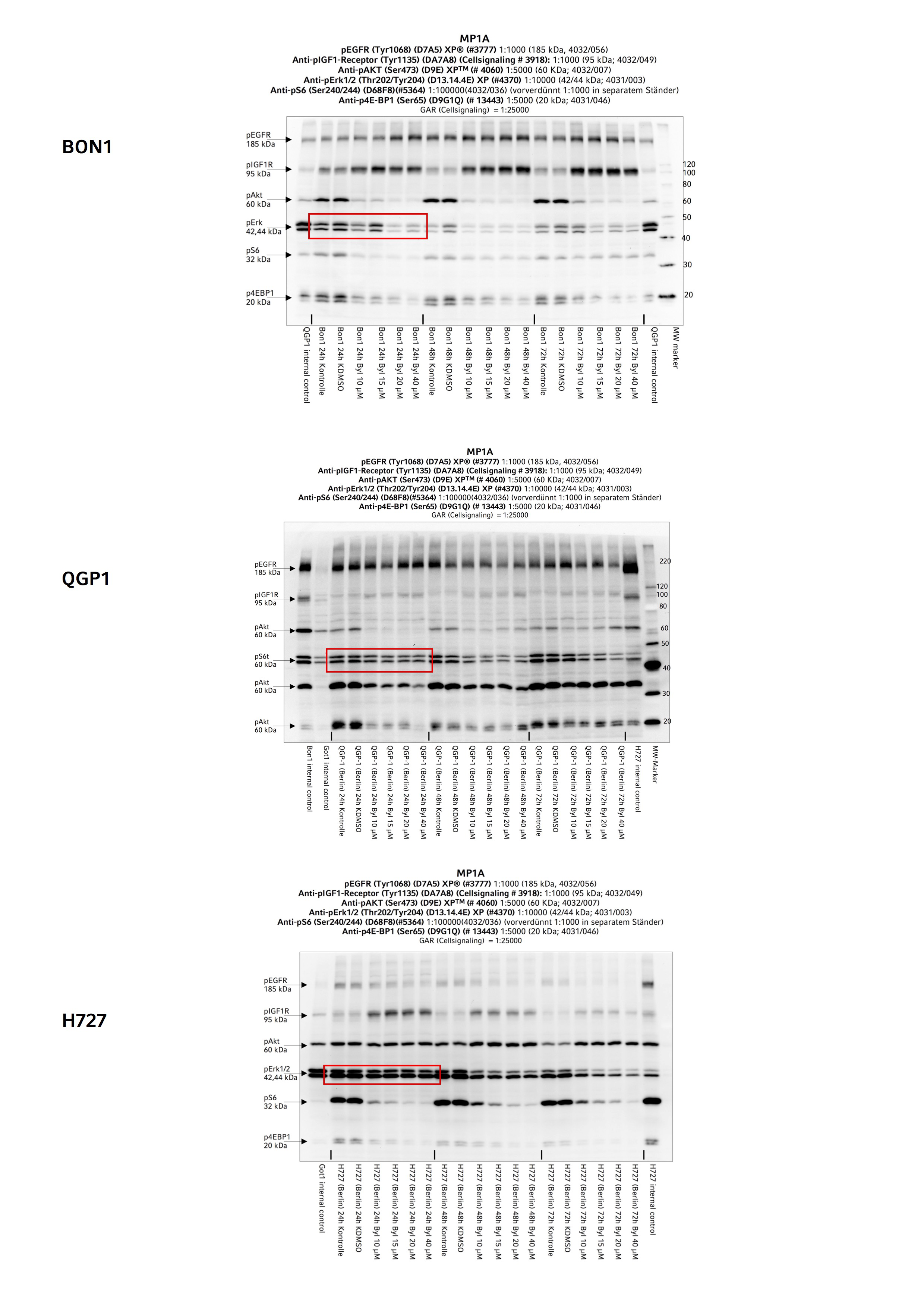

Supplement: S17 Fig — (TIF) [file pone.0182852.s017.tif]

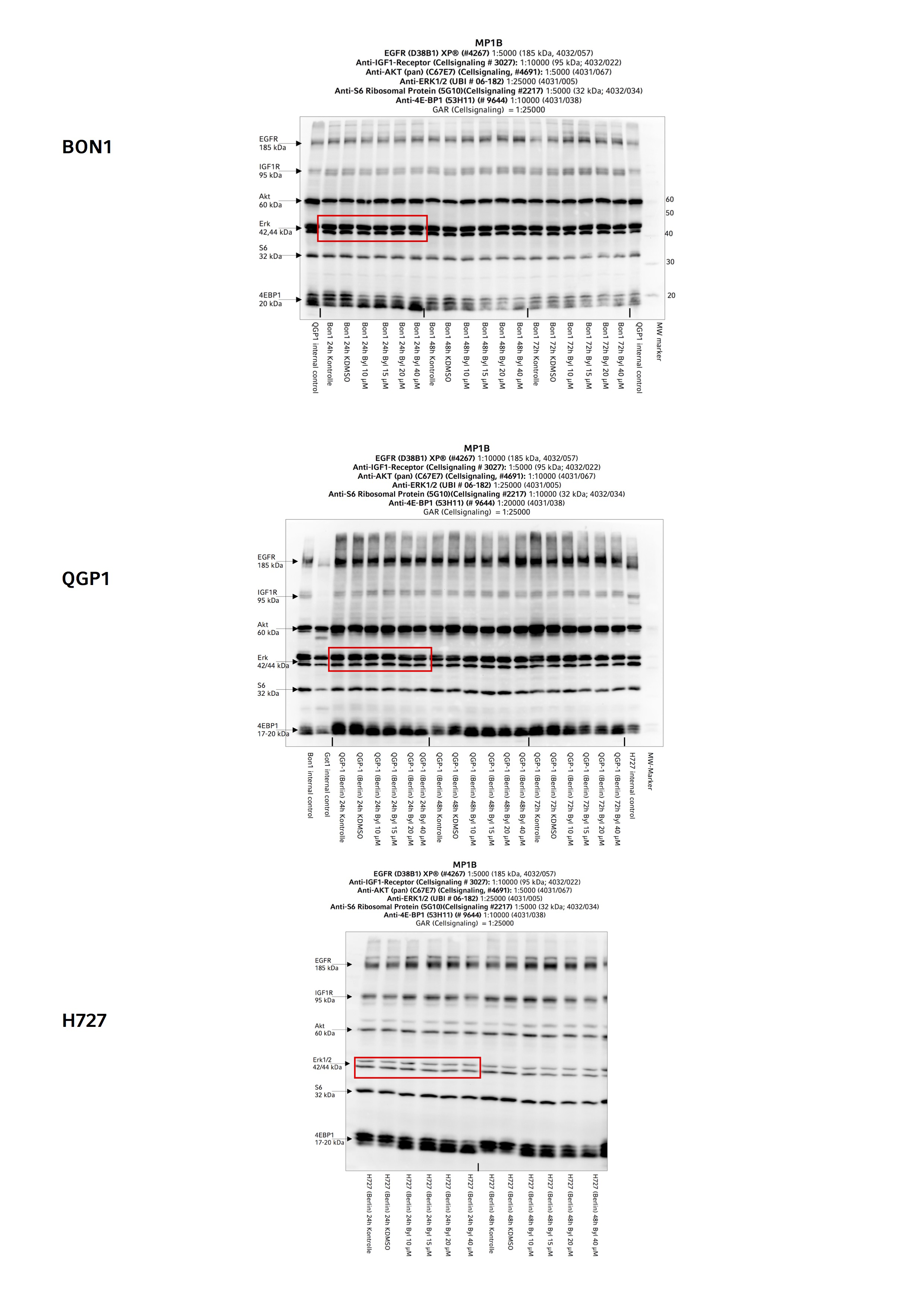

Supplement: S18 Fig — (TIF) [file pone.0182852.s018.tif]

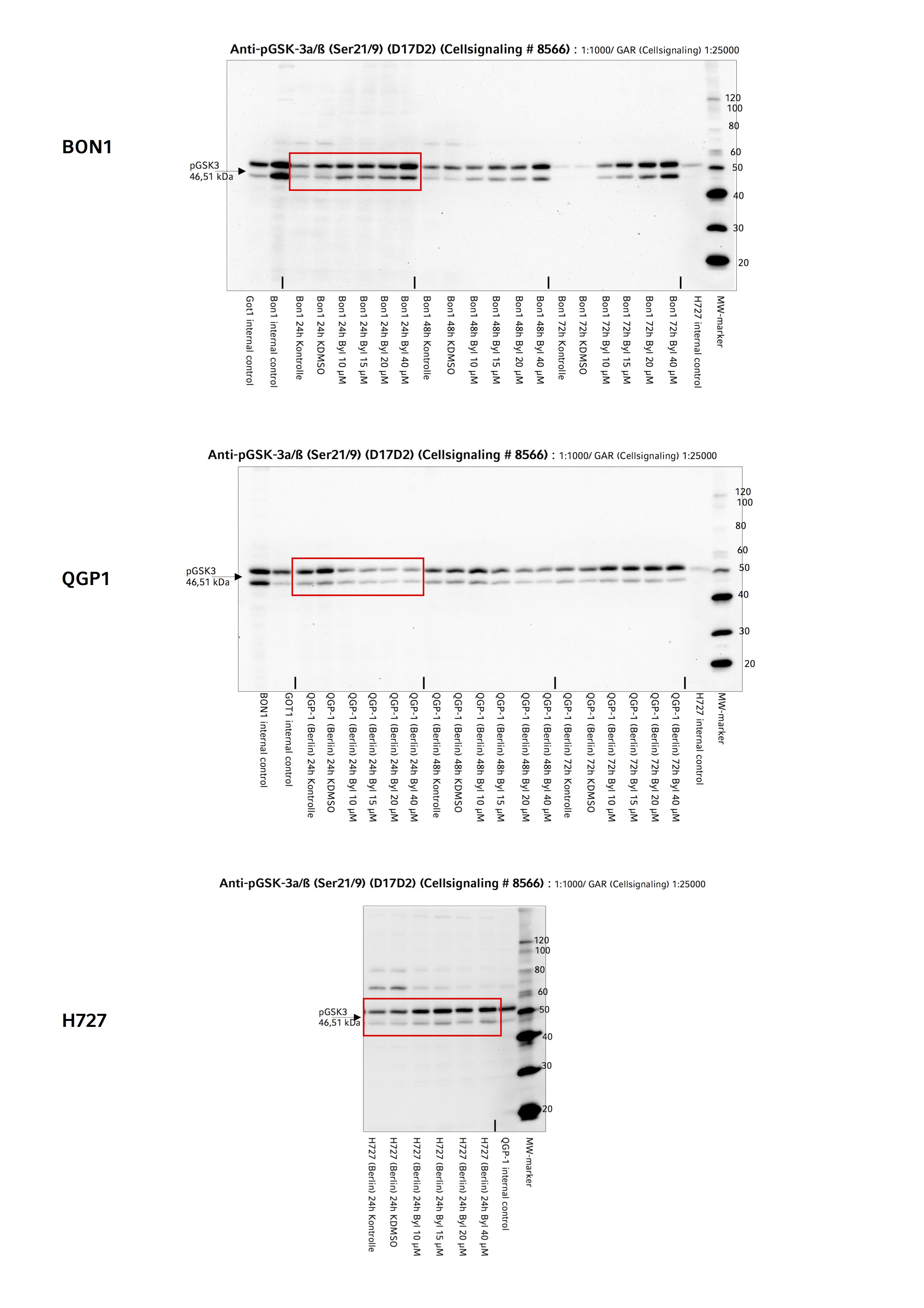

Supplement: S19 Fig — (TIF) [file pone.0182852.s019.tif]

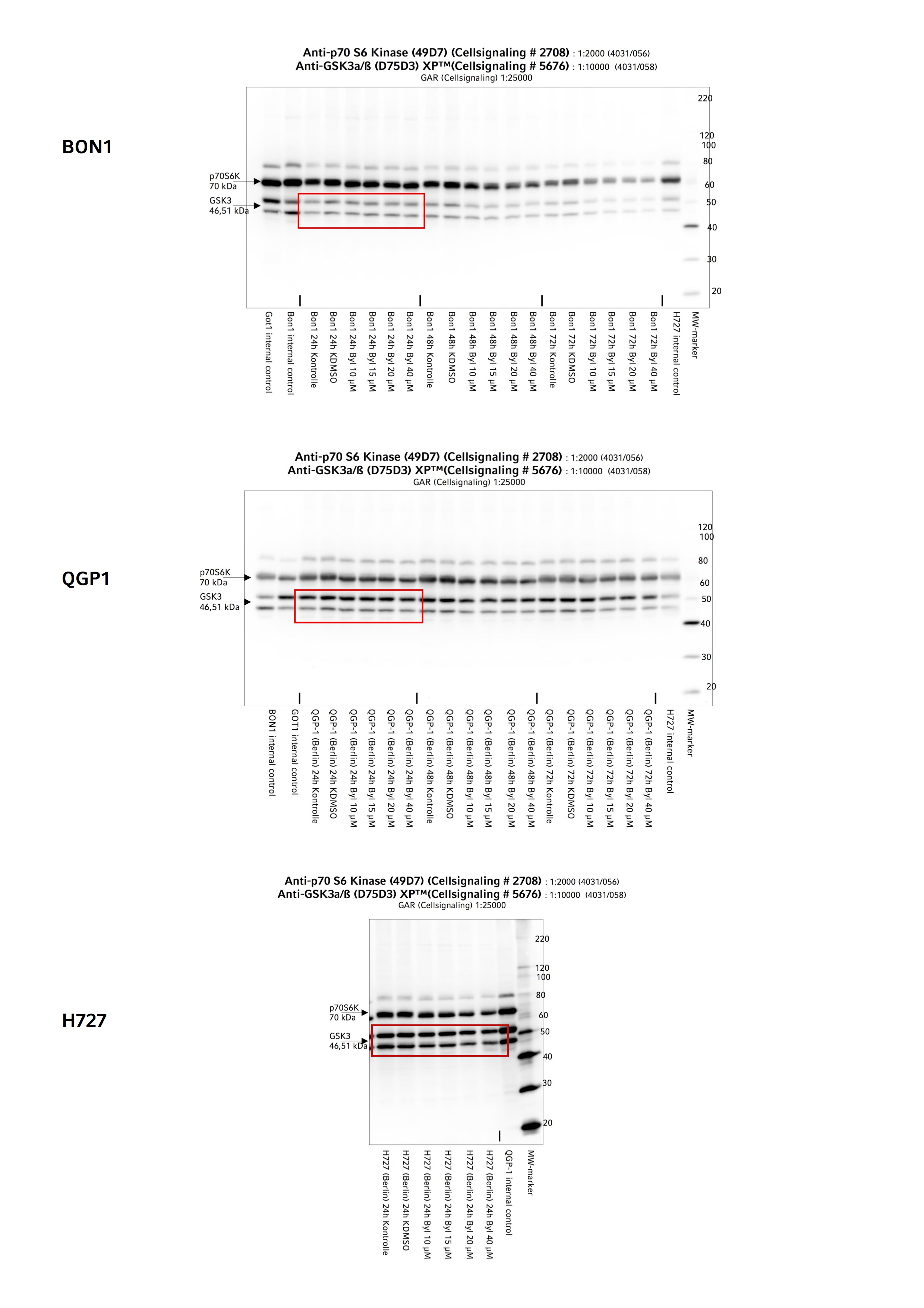

Supplement: S20 Fig — (TIF) [file pone.0182852.s020.tif]

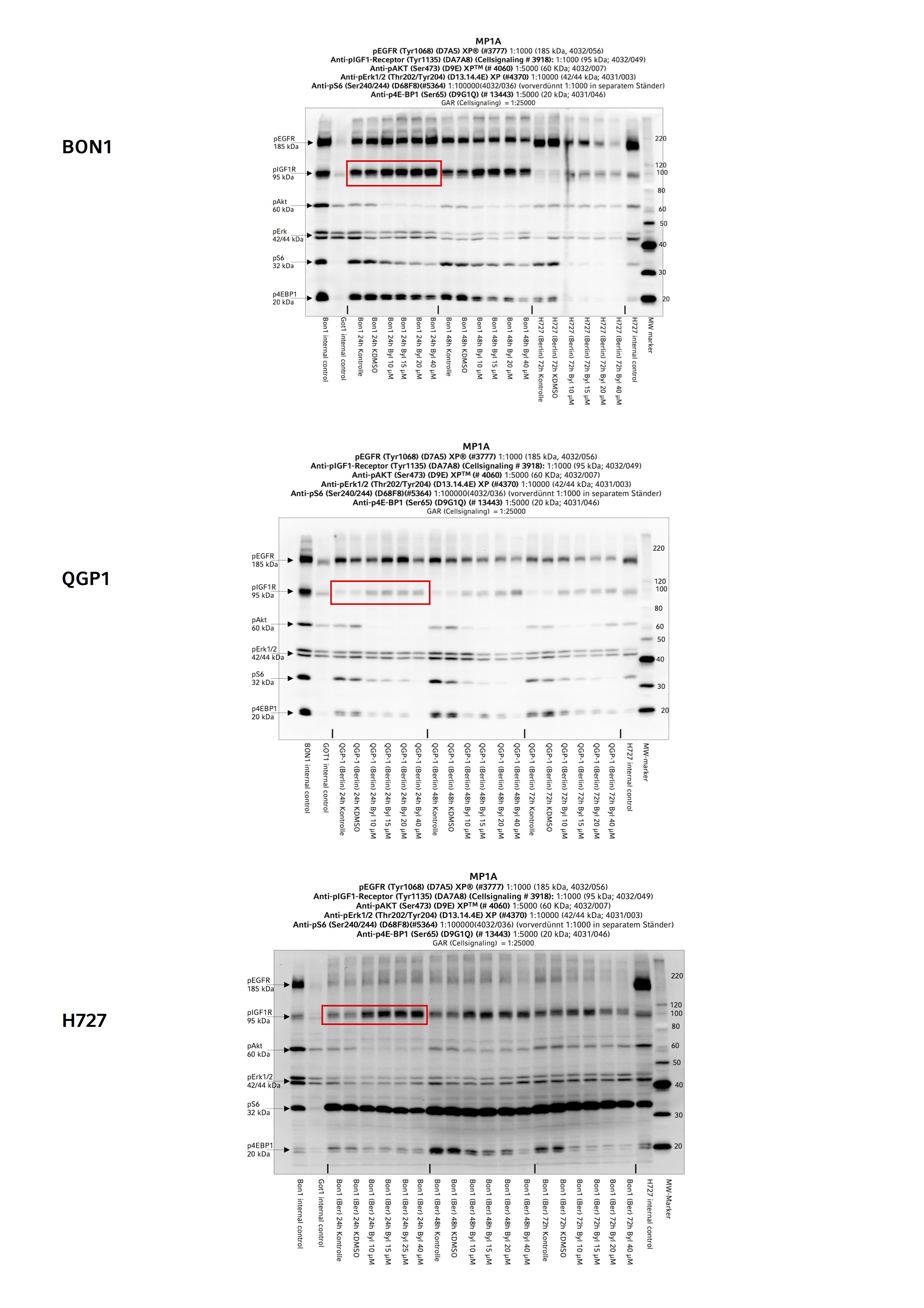

Supplement: S21 Fig — (TIF) [file pone.0182852.s021.tif]

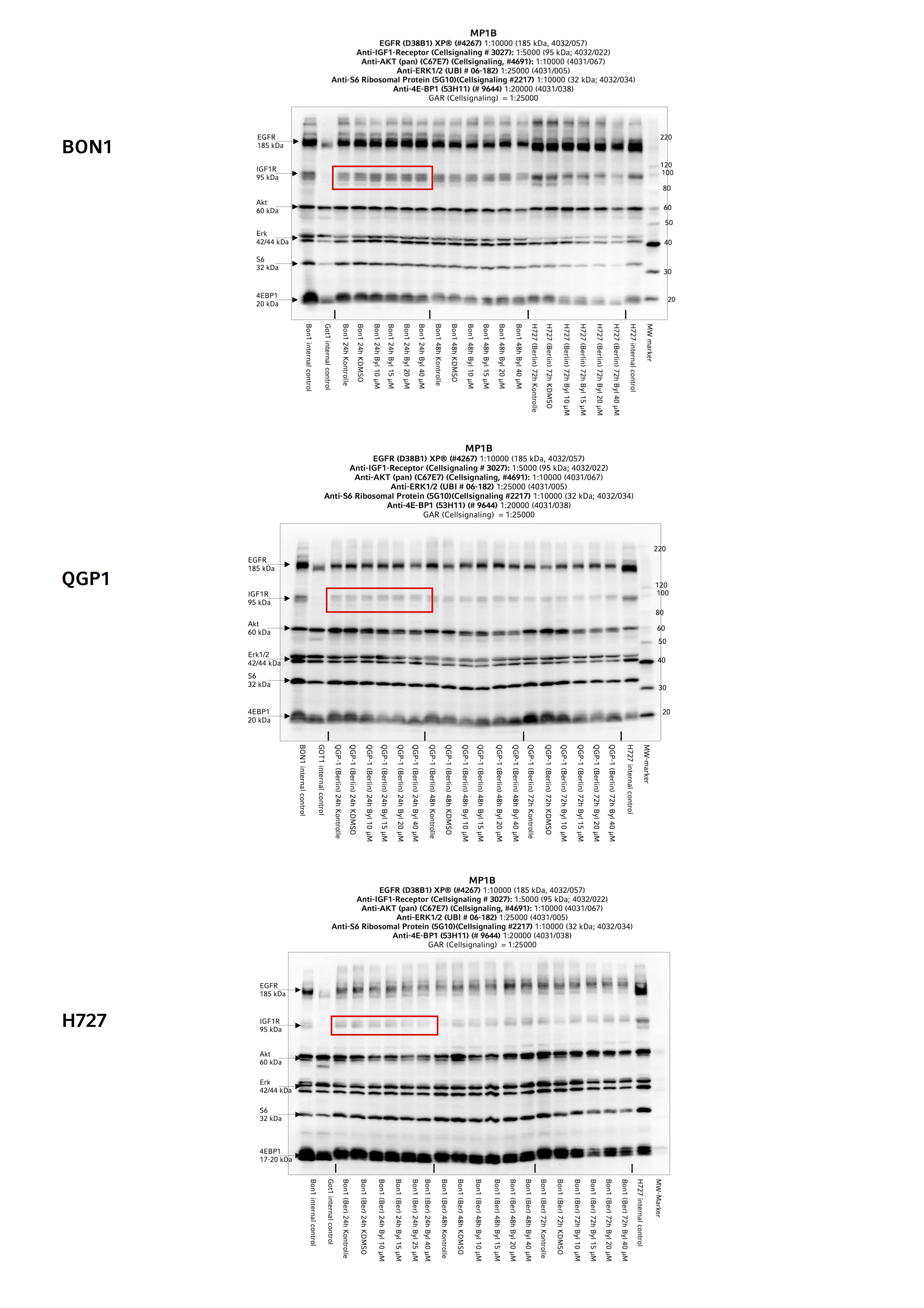

Supplement: S22 Fig — (TIF) [file pone.0182852.s022.tif]

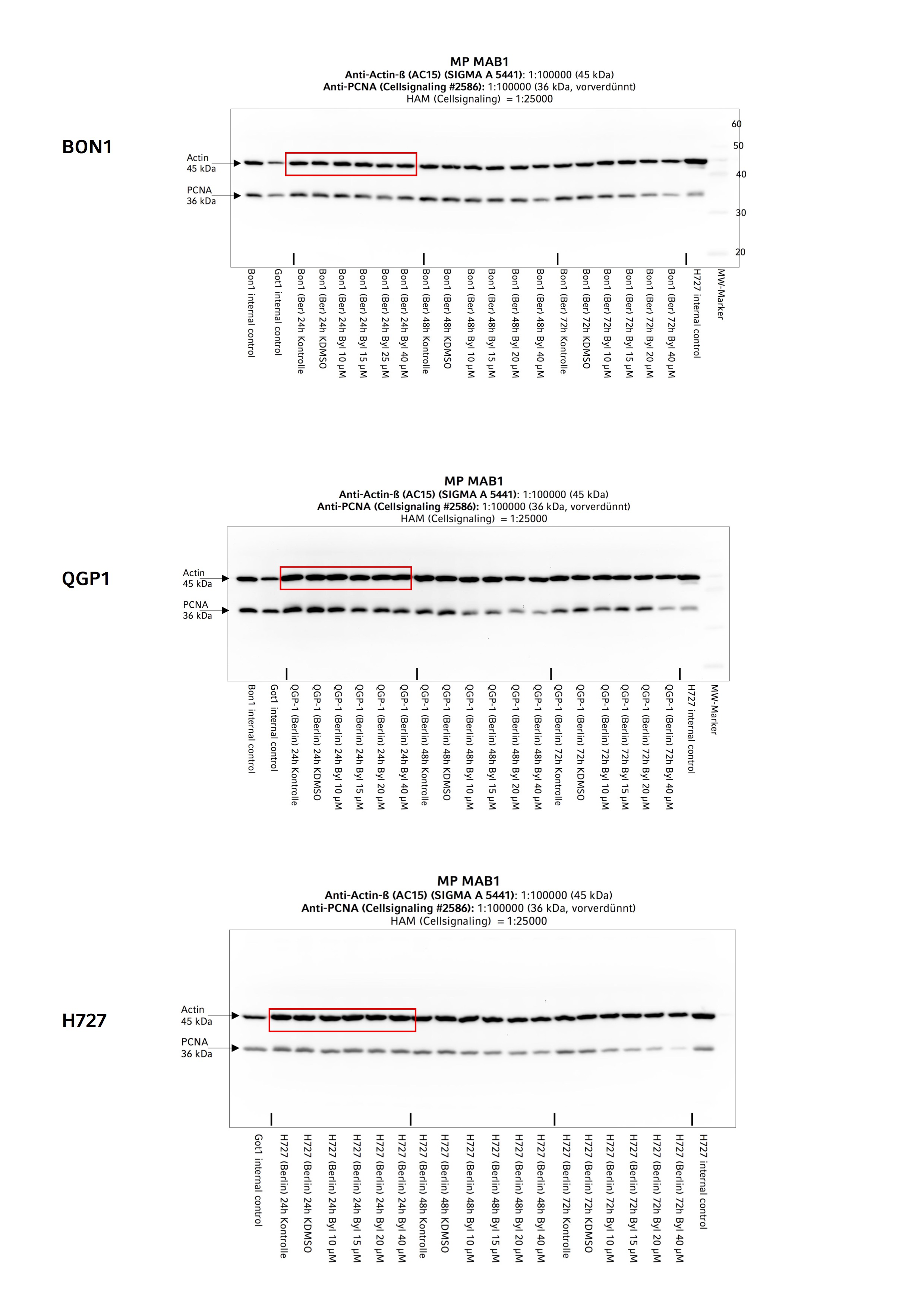

Supplement: S23 Fig — (TIF) [file pone.0182852.s023.tif]

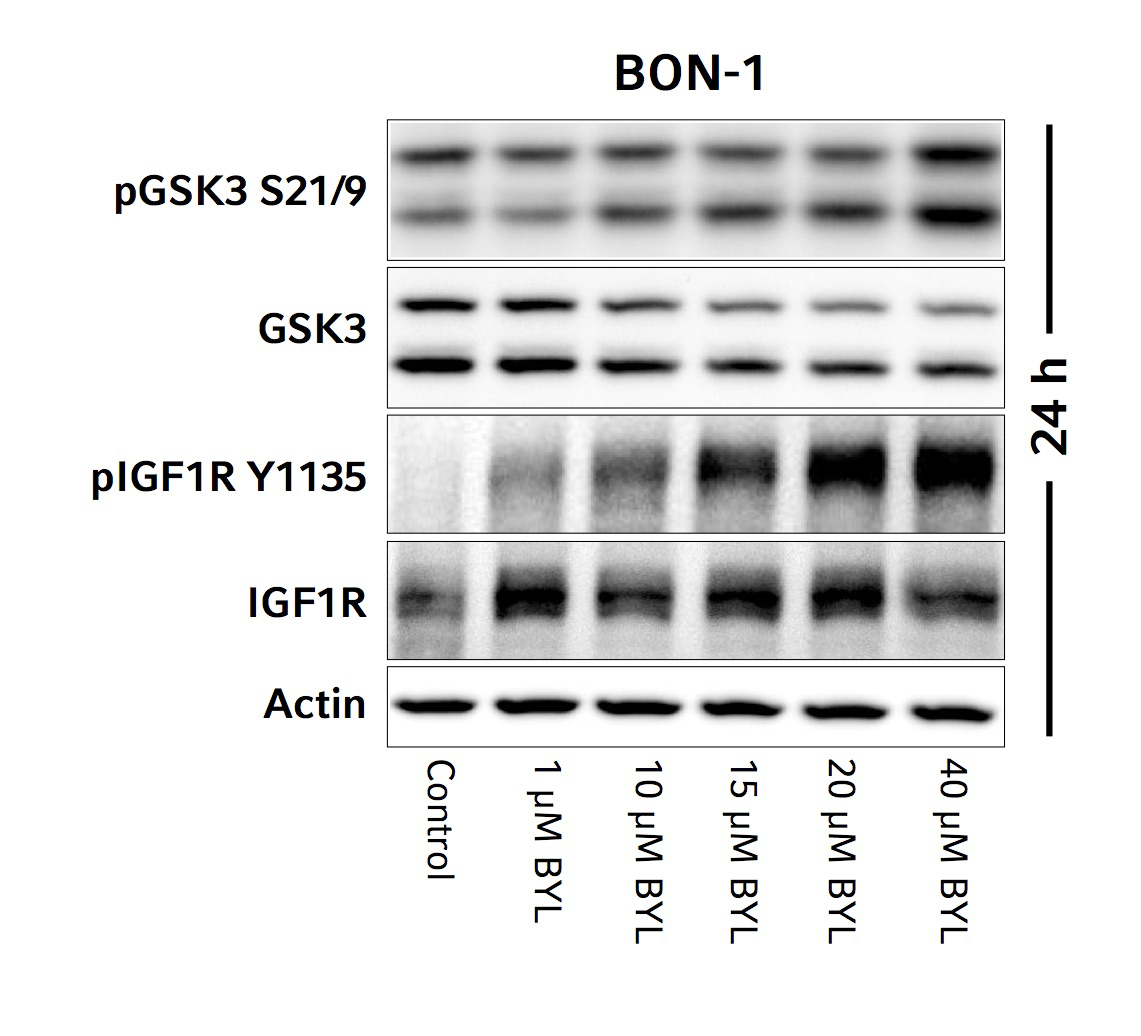

Supplement: S24 Fig — (TIF) [file pone.0182852.s024.tif]

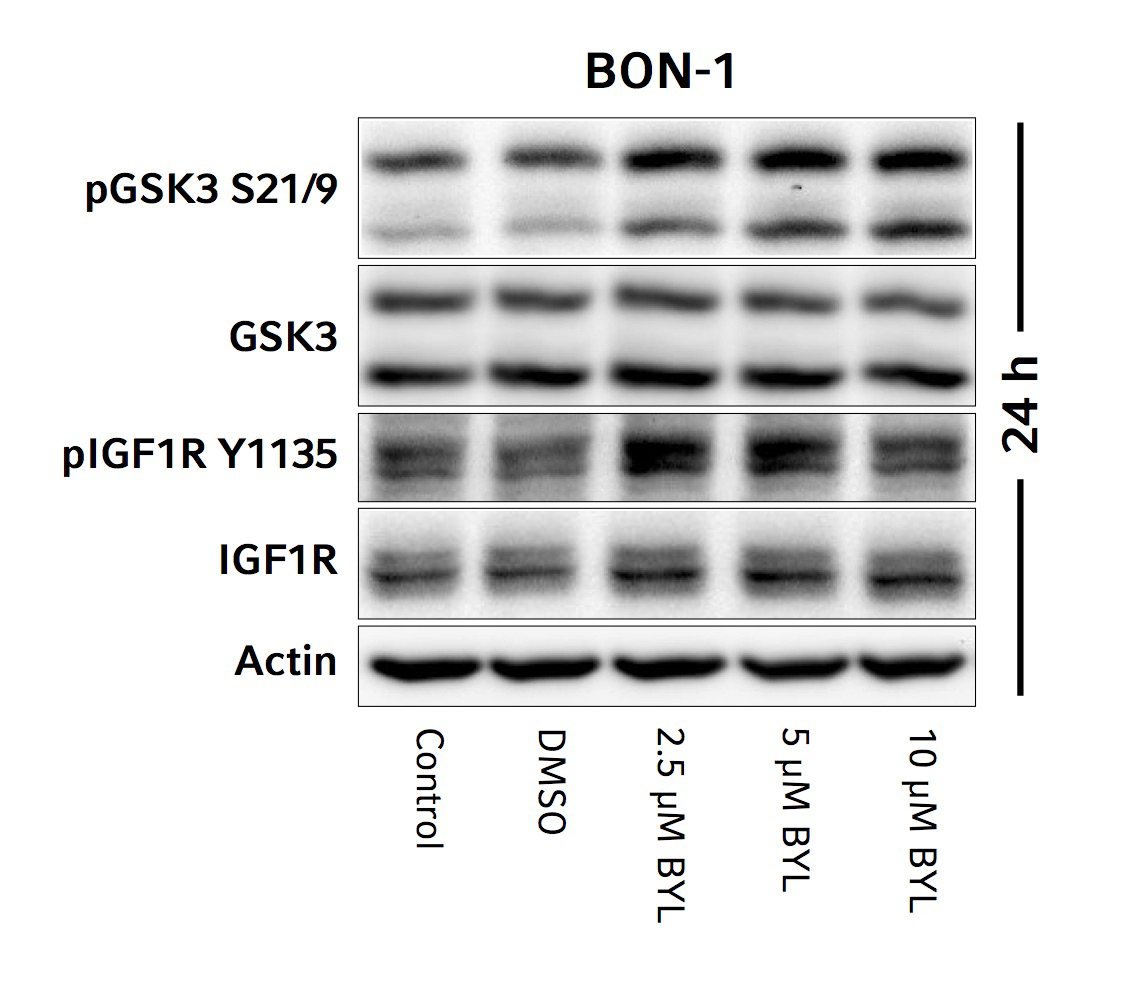

Supplement: S25 Fig — (TIF) [file pone.0182852.s025.tif]

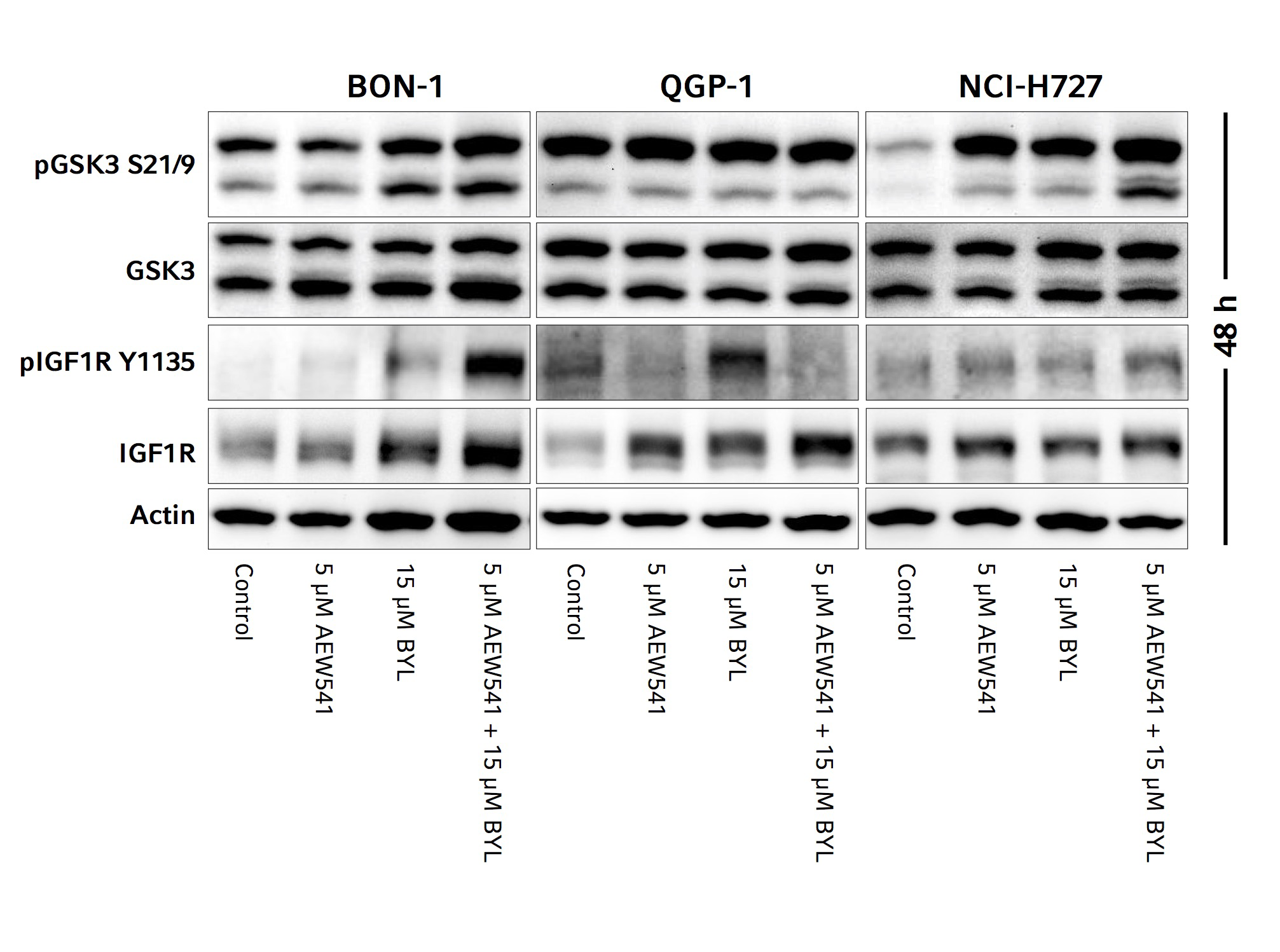

Supplement: S26 Fig — (TIF) [file pone.0182852.s026.tif]

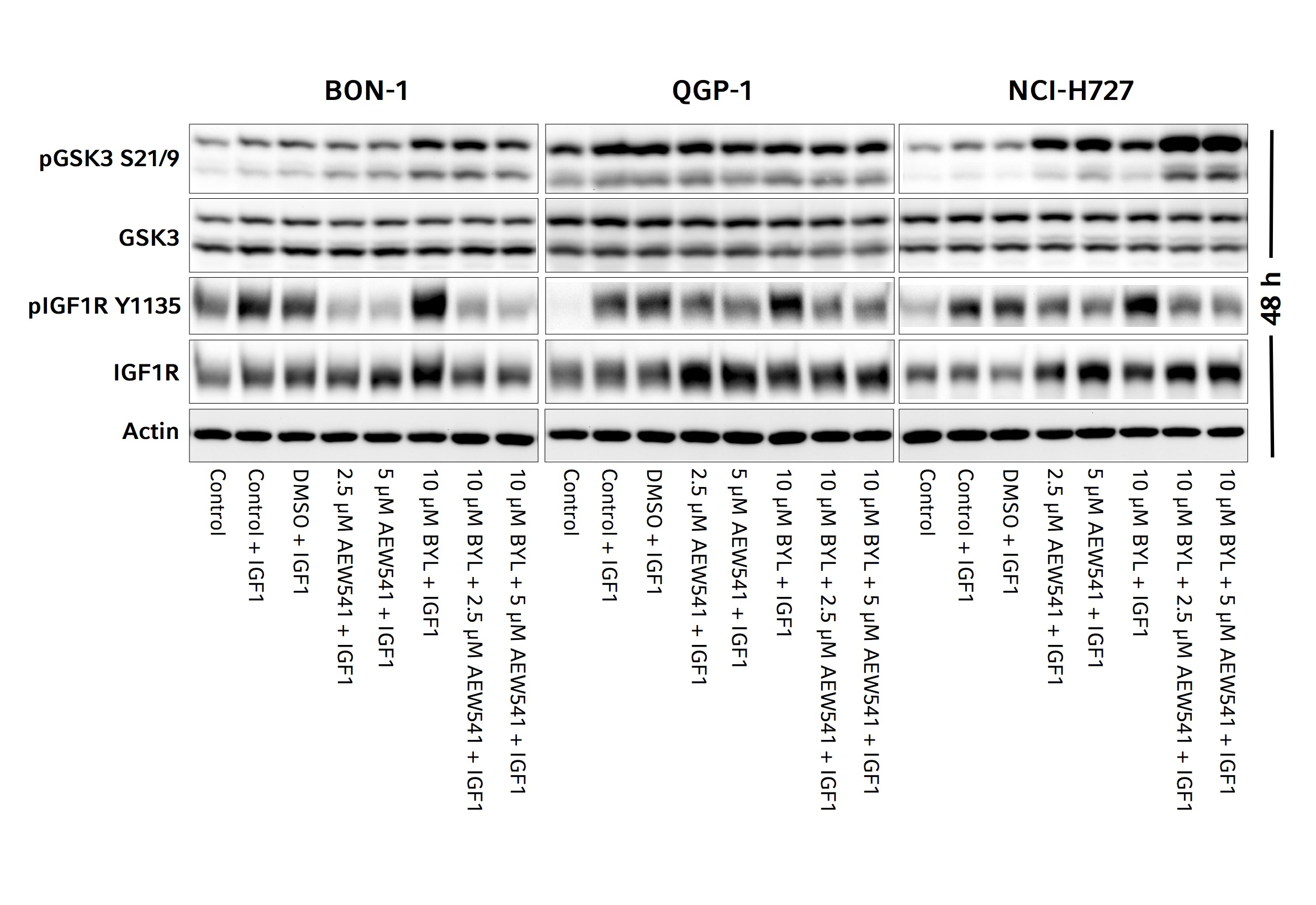

Supplement: S27 Fig — (TIFF) [file pone.0182852.s027.tiff]

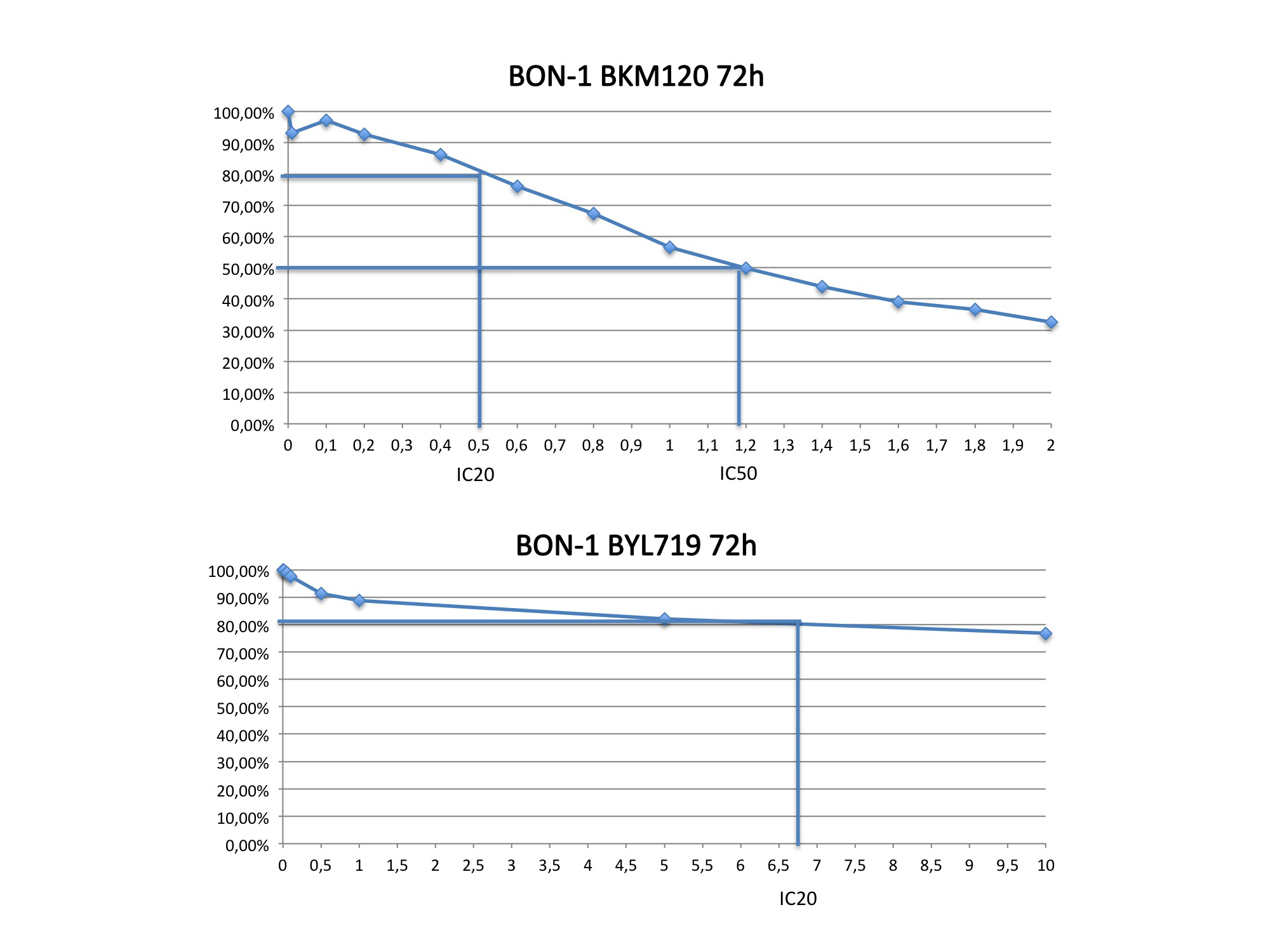

Supplement: S28 Fig — (TIF) [file pone.0182852.s028.tif]

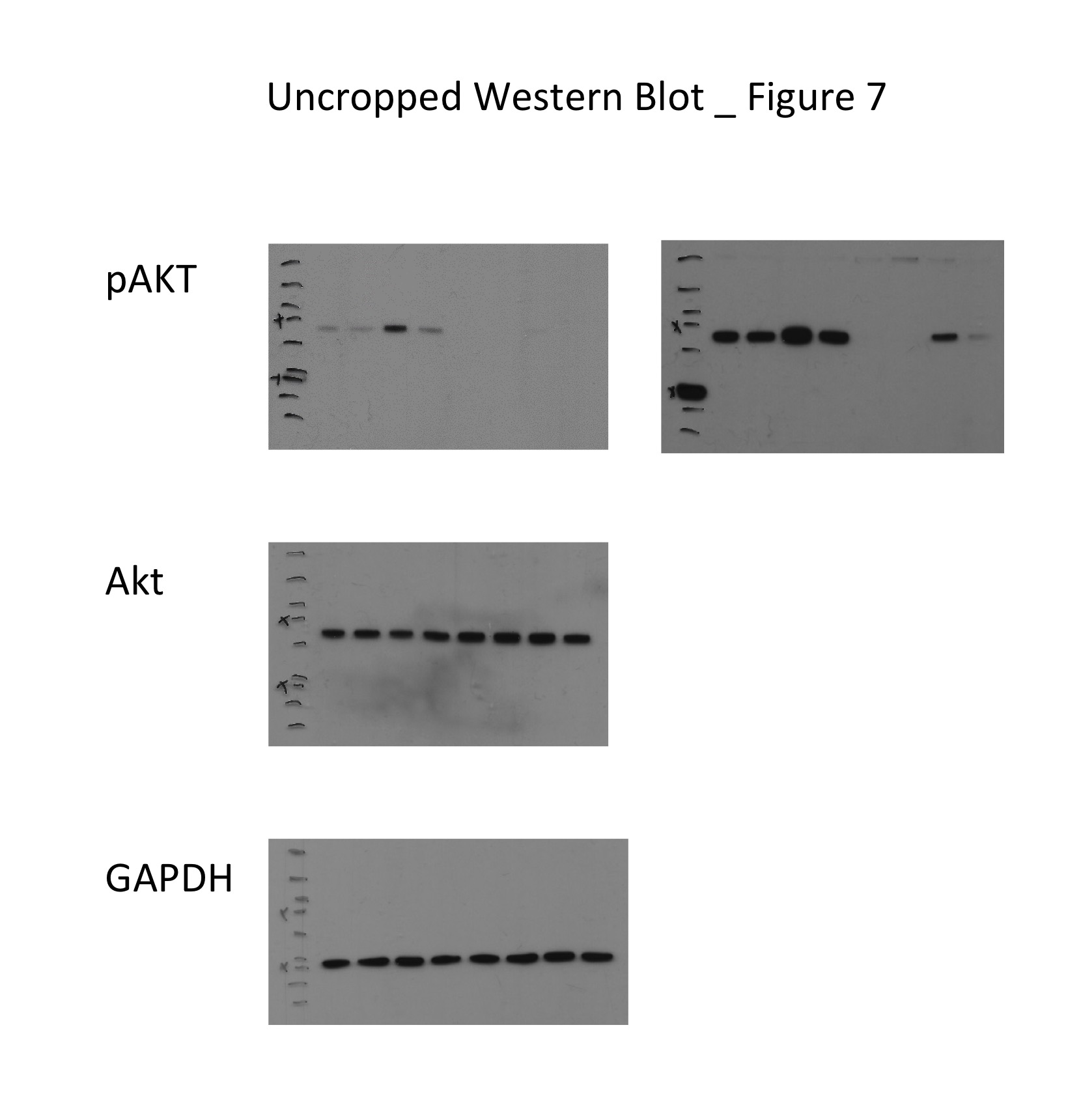

Supplement: S29 Fig — (TIF) [file pone.0182852.s029.tif]

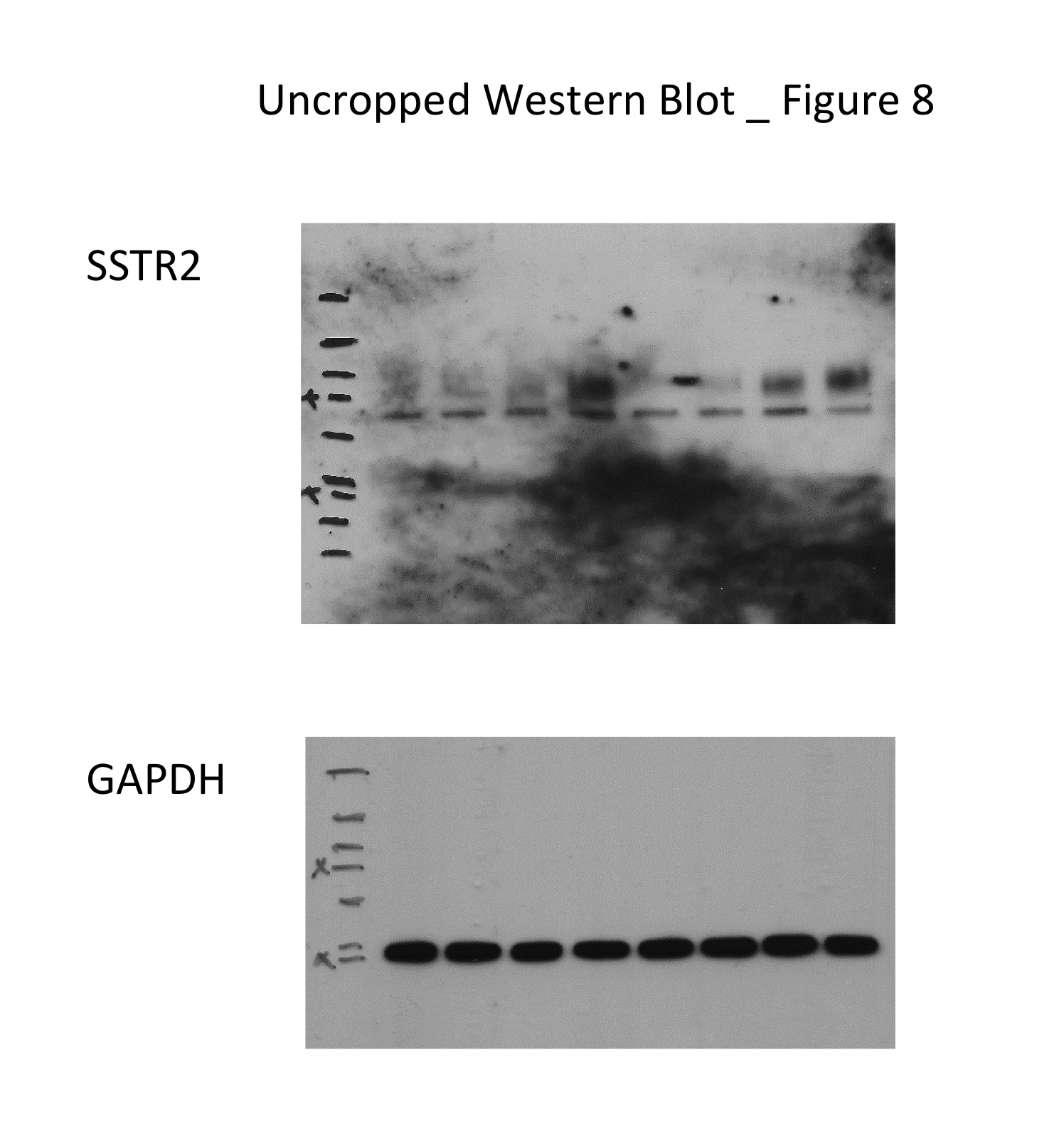

Supplement: S30 Fig — (TIF) [file pone.0182852.s030.tif]
